# Multimodal 3D-printed passive samplers to monitor, model and prioritise *in situ* pharmaceutical and pesticide pollution risks to an aquatic freshwater invertebrate, *Gammarus pulex*

**DOI:** 10.1039/d5em00452g

**Published:** 2025-10-14

**Authors:** Alexandra K. Richardson, Stephen Stürzenbaum, David A. Cowan, David J. Neep, Leon P. Barron

**Affiliations:** a MRC Centre for Environment & Health, Environmental Research Group, School of Public Health, Faculty of Medicine, Imperial College London 86 Wood Lane London W12 0BZ UK leon.barron@imperial.ac.uk; b Dept. Analytical, Environmental & Forensic Sciences, Institute of Pharmaceutical Sciences, School of Cancer and Pharmaceutical Sciences, Faculty of Life Sciences & Medicine, King's College London 150 Stamford Street London SE1 9NH UK; c Agilent Technologies UK Ltd Essex Road Church Stretton SY6 6AX UK

## Abstract

Calibrated 3D-printed multi-modal passive sampler devices (3D-PSDs) were used herein both to monitor contaminants of emerging concern (CECs) in freshwater and to estimate *in situ* chemical toxic and effect units for the aquatic invertebrate, *Gammarus pulex*, to support prioritisation strategies. A six-month study of water, biota, and 3D-PSDs in a heavily wastewater-impacted urban river catchment in London revealed 112 CECs detected, including pesticides, pharmaceuticals, illicit drugs and transformation products (water = 50; 3D-PSDs = 99; and *G. pulex* = 58 CECs). In *G. pulex*, the top three most concentrated CECs were citalopram (an antidepressant, at 101 ± 11 ng g^−1^), imidacloprid and clothianidin (both neonicotinoid pesticides, at 63 ± 12 and 52 ± 39 ng g^−1^, respectively). Principal component analysis revealed that passive sampler data represented chemical occurrence in the *G. pulex* better than using water data. Strong correlations existed between the passive sampler and biomonitoring data (*R*^2^ > 0.84, *p* < 0.05) indicating a possibility to infer risk from the device directly and without using calibrated PSD uptake rates (*R*_s_). This new approach showed promise as a potentially cost-effective way to rapidly prioritise sites and CECs for large-scale risk assessment campaigns for these species.

## Abstract 2

Multimodal 3D-printed passive sampler devices (3D-PSDs) were used to monitor contaminants of emerging concern (CECs) in freshwater and to estimate chemical toxic and effect units in a benthic invertebrate.

## Graphical abstract

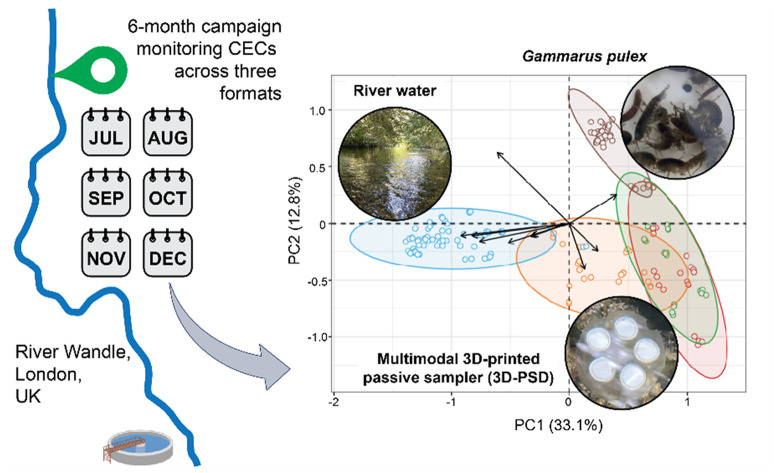

**Environmental significance**

Our study introduces a new multimodal 3D-printed miniaturised passive sampler-based model as a more ethical and scalable tool for chemical risk prioritisation in a benthic invertebrate, *Gammarus pulex*. It presents. Uptake rate (*R*_s_) calibration data for 93 chemicals of emerging concern on anion, cation and neutral sorbent chemistries. A six-month monitoring campaign in a wastewater-impacted river that identified 117 CECs in water, passive samplers and *G. pulex*. Principal component analysis showing passive samplers represented chemical occurrence in *G. pulex* better than water samples. A strong, linear correlation existed between the risk estimated using the 3D-PSD and biomonitoring data (*R*^2^ > 0.84, *p* < 0.05). The rising risks of the neonicotinoid imidacloprid in urban UK rivers following blanket prophylactic use as a pet parasiticide.

## 1. Introduction

Over 350 000 chemicals have been inventoried for manufacture and use with an estimated 220 billion tons of chemicals released into the environment per annum.^1,2^ Thousands of unregulated ‘contaminants of emerging concern’ (CECs) have been detected to date in various environmental matrices.^3–6^ Given the multifaceted scale of chemical water contamination, knowledge of causal impacts on aquatic wildlife and ecosystem function remains limited. For aquatic macroinvertebrates in particular, even less is known,^7,8^ which is concerning given their key roles in nutrient cycling, improving water quality, and the potential for biomagnification through food webs.^9^

Of relevance to this study, a wastewater-impacted urban freshwater environment, both pesticides and pharmaceuticals are of particular concern to invertebrates due to the effects they can elicit.^10^ Exposure to these and other everyday-use chemicals have been shown to arise from agricultural and water treatment practices, storm runoff, leaching and (un)intentional release. Indirect impacts on invertebrates have also been reported, including disruption of predator–prey interactions^11^ and species abundance through selective pressure of pesticides on invertebrates.^12^ Directly measuring CEC concentrations in biota tissues is the best method of determining long-term exposure. However, practically measuring CECs in wild-caught biota can be challenging in terms of sampling and standardising specimens for age, size, moult cycle, maturity and sex.^13,14^ In addition, the sample preparation is costly, complex and labour-intensive, often limiting large-scale application. As a result, for many CECs, monitoring exposure in biota is usually performed by using water or sediment, giving insights as to the route of exposure.^15^

Advanced computational tools for comparative and predictive toxicology have emerged, which use such monitoring data to help with CEC prioritisation and to estimate the effects in and across species (*e.g.*, the US EPA CompTox now includes information on >1 million chemicals.^16^ However, the use of water or sediment analysis may fundamentally limit the relevant chemical space coverage, as it may not represent the full extent of CECs present in biota, especially at very low concentrations.^15,17,18^ This may introduce some element of bias in the prioritisation workflow by focusing on those compounds that are more easily detectable rather than those that have the greatest impact. Additional methods are needed that are more cost-effective, sustainable and convenient to work at large scales, but also can be standardised and enable better detection of more compounds at lower concentrations over time. In addition, such new approaches should aim to improve knowledge of CEC impacts in biota without a fundamental requirement for animal sampling and/or sacrifice.

Passive sampling is an alternative technique for monitoring CECs in water. It is based on the flow of analytes from the environment to a receiving sorbent along a diffusion gradient that mathematically follows first-order uptake kinetics.^19^ After calibration, it enables measurement of the time-weighted average (TWA) concentrations of dissolved contaminants *in situ* over the deployment period. As an accumulative process, passive sampling often enables easier analytical measurement of more analytes compared to grab sampling,^20^ not least as the latter only represents the contaminants present at the exact time of sampling. Passive sampling has also been suggested as a potential ‘proxy’ for biota in environmental monitoring studies due to their shared accumulative nature, with chemical concentrations in passive samplers correlating with concentrations measured in biota.^21,22^ In addition, passive samplers have negligible background contamination, can be deployed in aquatic environments unable to support biota, and can be standardised, enabling comparisons across study systems.^13^ Such use of passive samplers could potentially be used in a variety of applications and requires relatively simpler extraction procedures than biota samples.

We recently developed a new low-cost 3D-printed passive sampler device (3D-PSD) that was shown to be suitable for large-scale risk assessment in water for >100 pharmaceuticals, illicit drugs, pesticides and their transformation products.^23^ This current work aimed to investigate whether such devices could be used to estimate *in situ* CEC occurrence in biota. The first objective was to understand CEC occurrence in a wastewater-impacted, urban and chalk-fed river (River Wandle, London, UK) using 3D-PSDs configured with multiple sorbents. The next objective was to compare 3D-PSD concentration data with CECs concentrations measured in grab water samples and samples of *G. pulex* taken over the same period to determine if any relationship was present and to assess risk across all matrices. The data was then used to build a new model to assess CEC risk to *G. pulex* in this river. This is the first study to document the successful combination of small multimodal passive sampling tools, rapid chemical analysis of water samples and biota extracts for *in situ* risk assessment of large numbers of CECs in a river catchment that could then be applied at large scale internationally.

## 2. Materials & methods

### 2.1 Reagents, chemicals, and consumables

Organic solvents including methanol (MeOH), acetonitrile (MeCN), and propan-2-ol (IPA) were purchased from VWR International Ltd (Lutterworth, UK). Formic acid (LC-MS grade) was acquired from Millipore (Bedford, USA). All reagents were of analytical grade or higher. For compound identification and quantification purposes, a standard mix of 200 compounds (*n* = 164 analytical standards and *n* = 36 stable isotope-labelled internal standards (SIL-IS), purity ≥97%) was used. The full list of compounds is reported in the SI S1. All controlled substances (*e.g.*, Class A illicit drugs) were acquired and used under a UK Home Office license.

As per OECD Test Guidelines,^24^ salts used for the preparation of AFW included magnesium sulphate (MgSO_4_) (Fisher Scientific, Leicestershire, UK), sodium hydrogen carbonate (NaHCO_3_) and potassium chloride (KCl) (Alfa Aesar, Massachusetts, USA), and calcium chloride (CaCl_2_) (ACROS Organics, Geel, Belgium). Salt stock solutions were diluted to the required concentration using ultrapure water dispensed from a 18.2 MΩ cm Millipore Milli-Q water purification system (MilliporeSigma, Massachusetts, USA).

### 2.2 3D-printed passive sampler device (3D-PSD) procedures

The 3D-PSD housing was fabricated using an Asiga MAX mini 3D-printer (Puretone™ Ltd, Kent, UK) with a commercially available methacrylate-based resin (PlasCLEAR V2) to provide a low-cost, on-demand supply of passive samplers for this work. The planar design is similar to other commercially available devices, but the new design allows for multiplexing different sorbents within the same unit. The full design, development, and characterisation of the 3D-PSD has been described previously, including the prototype computer-aided design files, and details of its manufacture, assembly, calibration, deployment protocols, and validation reports.^23^ In brief, the 3D-PSD houses five separate 9 mm sorbent disks and consists of two core components, a top and a base, with a removable field-transport cap to protect the sorbent disks from contamination. The printed housing was non-porous, exhibited low leaching and is stable in a range of organic/aqueous solvents at acidic-alkaline pH.^25^

Three different sorbents were used in this work to capture a wide range of contaminants, including ionised molecules such as zwitterions,^26,27^ while still aligning with past passive sampler studies.^20,23,28^ The selectivity of these sorbents covers a broad range of mid-polarity chemistries, which are of relevance to biological uptake mechanisms in *G. pulex*.^29,30^ The neutral PSD phase was made from a hydrophilic–lipophilic balanced (HLB) type material poly (styrene–divinylbenzene (PS-DVB) with pyrrolidone moieties (AttractSPE® Disks HLB, Affinisep Val de Reuil, France)) to capture a range of bioavailable chemical contaminants.^28,31,32^ Functionalised HLB ion exchangers for mixed-mode (MM) retention of anions and cations were also used using AttractSPE® Disks Anion exchange—SR (PS-DVB-amine (MM-anion)) and AttractSPE® Disks Cation exchange—SR (PS-DVB-sulfonate (MM-cation)) to increase the sampled chemical space.^33,34^ The lower limits of detection (LODs) and other performance metrics for each analyte on each sorbent are presented in the SI (MM-anion and MM-cation) and previously published work (Richardson *et al.*, 2022). Supor poly (ether sulfone) (PES) membranes (0.2 μm, Pall Europe Ltd, Portsmouth, UK) were prepared for deployment as described previously.^23^

Sorbent disks and PES membrane were cut to 9 mm diameter using a cleaned 9 mm leather punch. Sorbent disks were conditioned with 5 mL of MeOH, followed by 5 mL of ultrapure water. Residual manufacturing residues were removed from the PES membranes with two sequential 24-hour washes with MeOH. To assemble the devices, a PES membrane was fitted inside each ‘well’ of the top component of the housing, followed by a sorbent disk, before the base component was used to sandwich them together. Refer to Richardson *et al.*, 2022, for full instructions.^23^ Assembled devices were stored submerged in ultra-pure water before deployment for a maximum of 48 hours to keep them hydrated and to avoid any interference from microbial growth. This ensured sorbents were fully wetted when deployed in the environment, increasing the accessibility for CECs to interact optimally with the sorbent chemistry.

### 2.3 Site selection and sample collection

The River Wandle is a freshwater urban chalk stream that originates in Croydon (one of the southern extremity areas of Greater London, UK) from an underground aquifer and flows through the London boroughs of Sutton and Merton before joining the River Thames in Wandsworth, Central London. Approximately 800 000 to 1 million people live in its catchment, and most of its length is accessible to the public. The study site (Ravensbury Park, 51.395227; −0.175981) was chosen based on earlier work showing CEC contamination at the site and is located roughly 1.5 km downstream from the discharge point of the local wastewater treatment plant (WWTP).^23^ The site was considered suitable due to good visibility, safe access, and the presence of a substantial *G. pulex* population across all months with no other *Gammarid* species present at the study site. In the River Wandle, *G. pulex* play an important role in food webs by consuming detritus and algae, recycling nutrients and serving as prey for fish. Importantly, they can tolerate pollution, making them an excellent organism to study chemical bioaccumulation.

For six months (July to December 2021, summer to winter), and on a monthly basis, ten 3D-PSDs were deployed at the selected study site for seven days. Refer to S2 for details of the deployment and retrieval protocol. This study period was chosen to capture a range of seasons and weather conditions that could impact the concentration of contaminants in the River Wandle. Flow data exists for the River Wandle across the timeframe studied at two active monitoring stations, above and below the WWTP and the study site (accessible at the National River Flow Archive^35^). At the South Wimbledon site (∼2.8 km downriver of the study site), the mean flow was 2.1 ± 0.7 m^3^ per day with the highest and lowest monthly flows recorded in October (2.7 ± 1.4 m^3^ per day) and September (1.8 ± 0.4 m^3^ per day), respectively.

*Gammarus pulex* samples were collected by kick-net sampling three to four times per month at the deployment and retrieval of the 3D-PSDs from the same location where the 3D-PSDs were deployed. Additional samples were collected mid-deployment and once outside of the deployment period. Refer to Table S1 for a timeline of sample collection and S2 for additional details regarding invertebrate sample collection. Water samples were concurrently collected with *G. pulex* samples using pre-rinsed 30 mL Nalgene^®^ bottles (Sigma-Aldrich, UK).

### 2.4 Sample preparation

#### 2.4.1 3D-PSDs samples

All 9 mm sorbent disks (including the field and a laboratory extraction blank) were extracted as per the protocol described in Richardson *et al.* with some slight alterations and modified elution solvent depending on the chemistry of the sorbent disks as per the manufacturer's guidelines.^23^ The elution solvents were MeOH, MeOH with 3% formic acid, and 5% ammonium hydroxide in MeOH for the HLB, MM-anion, and MM-cation sorbent phases, respectively. Refer to S3 for additional details regarding 3D-PSD sample extraction and preparation for LC-MS/MS analysis.

#### 2.4.2 *Gammarus pulex* samples

Before the first sampling timepoint, gammarids (*n* = 3) from the site were morphologically identified (taxonomic key from the Freshwater Biological Association^36^) and genotyped to confirm species identification as *G. pulex* (Fig. S1 and S4). Subsequent collections were not genotyped, but individuals were morphologically examined and confirmed to be *G. pulex* prior to extraction and analysis. Invertebrates sampled at each timepoint were processed as described previously, with some slight modifications,^37^ refer to S3 for details.

#### 2.4.3 Water samples

Water samples were prepared using previously published protocols.^20,23,38–41^ In short, 900 μL aliquots of river water were spiked with 100 μL of MeOH containing SIL-IS to a final concentration of 500 ng L^−1^. Quantification was performed using an external matrix-matched calibration curve (5 to 2000 ng L^−1^) prepared from a pooled sample containing equal volumes of water from the monthly collections. All water samples were vortexed before filtering through 0.2 μm syringe filter directly into a deactivated HPLC vial (Agilent A-Line, Agilent Technologies, Santa Clara, CA, USA).

### 2.5 Instrumental analysis

All samples were analysed using a rapid LC-MS/MS method performed on a LCMS-8060 instrument (Shimadzu Corporation, Kyoto, Japan) as previously reported for 164 CECs.^20,40^ Separations were performed using a short Raptor 5.0 × 3.0 mm, 2.7 μm biphenyl guard column (Restek, Pennsylvania, USA) with a flow rate of 0.5 mL min^−1^ and an injection volume of 10 μL. The elution program consisted of 10% of mobile phase B (MPB, 0.1% v/v formic acid in 50 : 50 MeOH : MeCN; MPA = 0.1% aqueous (v/v) formic acid) for 0.2 min, then a ramp to 60% MPB over 2.8 min and 100% MPB for 1 min followed by a re-equilibration period of 1.5 min for a total run-time of 6.5 min. Multiple reaction monitoring (MRM) was performed using at least two transitions per analyte.^40^ Data were acquired and processed using Shimadzu LabSolutions and LabSolutions Insights LCMS, respectively.

### 2.6 Calculation of toxic units, effect pressures and risk quotient

Risk quotients (RQ) in water were calculated as a ratio between the measured environmental concentration (MEC) and the predicted no-effect concentration (PNEC) taken from the NORMAN Ecotoxicological Database^42^ as per eqn (1). The thresholds for risk were assigned as: insignificant risk (<0.1), low risk (0.1–1), medium risk,^1–10^ and high risk (>10), aligning with previous research.^38,39^1
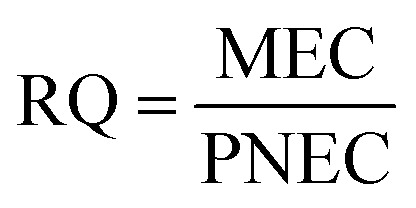


Internal toxic (TU_int_) and effect (EU_int_) units were calculated as described previously.^37,43^ The risk posed by pesticides was determined using toxic units (eqn (2)), where the concentration in the *G. pulex* tissues was divided by the internal EC50 value (EC50_int_, eqn (3)), calculated as the EC50 literature value (lowest 48 h acute exposure in *G. pulex* or *Daphnia magna*) multiplied by the bioconcentration factor (BCF) as estimated using either the EPI Suite BCFBAF v 4.11 (ref. 44) or a previously published BCF prediction model in *G. pulex*.^18^ The higher BCF values were used in calculations to estimate the worst-case scenario. For all pharmaceutical compounds that did not have a reported EC50 value, the EU_int_ were calculated using the predicted critical environmental concentration (PC_crit_) as described by Fick *et al.* (eqn (4), ref. 45).2
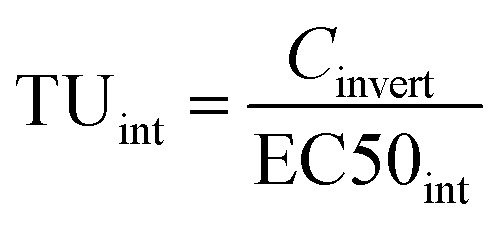

3 EC50_int_ = EC50·BCF

4
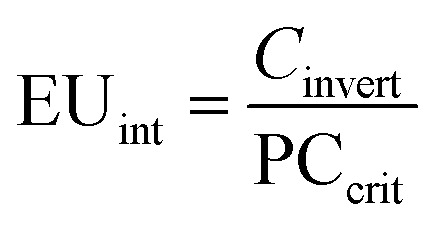


### 2.7 Data analysis and visualisation

Microsoft excel (v. 2402, Microsoft Corporation, WA, USA) was used for the initial analysis, curation and modelling.^46^ Python (v. 3.7.12, Python Software Foundation, DE, USA) packages ‘pandas’ and ‘NumPy’ were used for data handling and analysis, ‘scikit-learn’ was used for data preprocessing and scalar transformations, and ‘seaborn’ was used for data visualisations.^47–51^ Statistical analysis was performed using the ‘stats’ and ‘ggbiplot’ packages in RStudio (v. 576, Posit (PCB), MA, USA).^52,53^ For clustering analysis (Principal Component Analysis (PCA) and hierarchical clustering (HCA)), chemical data was scaled between 0 and 1 for each measurement replicate using scikit-learn's MaxMinScalar function.

## 3. Results & discussion

### 3.1 CEC occurrence in the River Wandle over six months

#### 3.1.1 CEC concentrations in water grab samples

In total, 50 unique compounds were detected in water samples (of which 41 were quantifiable) from 21 collection timepoints. CEC concentrations ranged from 10 ± 2 ng L^−1^ (atrazine, October 2021) to 1350 ± 55 ng L^−1^ (nicotine, October 2021), Fig. 1(a). Of these, 10 compounds (acetamiprid, atrazine, azithromycin, carbamazepine, clarithromycin, diclofenac, imidacloprid, sulfamethoxazole, terbutryn, and trimethoprim) are either priority pollutants or have been included in EU Water Framework Directive Watch Lists.^54^ All detected compounds were consistent with other studies from our group monitoring the River Wandle in 2020 and 2021,^23,39^ indicating a relatively consistent chemical profile. Importantly, these previous publications focused extensively on the spatial distribution of CECs in water samples in this river to identify the most contaminated sites. In particular, we previously showed elevated concentrations of several CECs in November–December in 2020 and 2021, resulting in cumulative RQs > 10 for all compounds at all sites beneath the WWTP outfall. This particular stretch of river also receives inputs from storm overflows and sewer misconnections at several locations.^38,39^ Therefore, the selection of Ravensbury Park enabled monitoring of the within-year temporal CEC flux as one of the most contaminated sites on the River Wandle, particularly to study the impact on invertebrate organisms with a life-cycle of approximately one year.

**Fig. 1:**
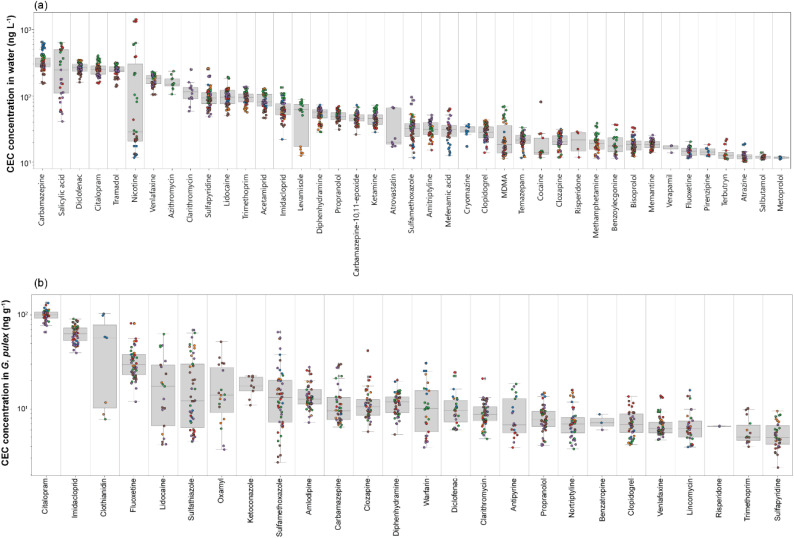
Boxplots representing the range of contaminant concentration in (a) freshwater, (b) *G. pulex* samples over six months. Coloured dots indicate the month of sample collection, blue = July, orange = August, green = September, red = October, purple = November, and brown = December. Refer to S2 for monthly data.

#### 3.1.2 Quantitation of CECs on 3D-PSDs

**Fig. 2:**
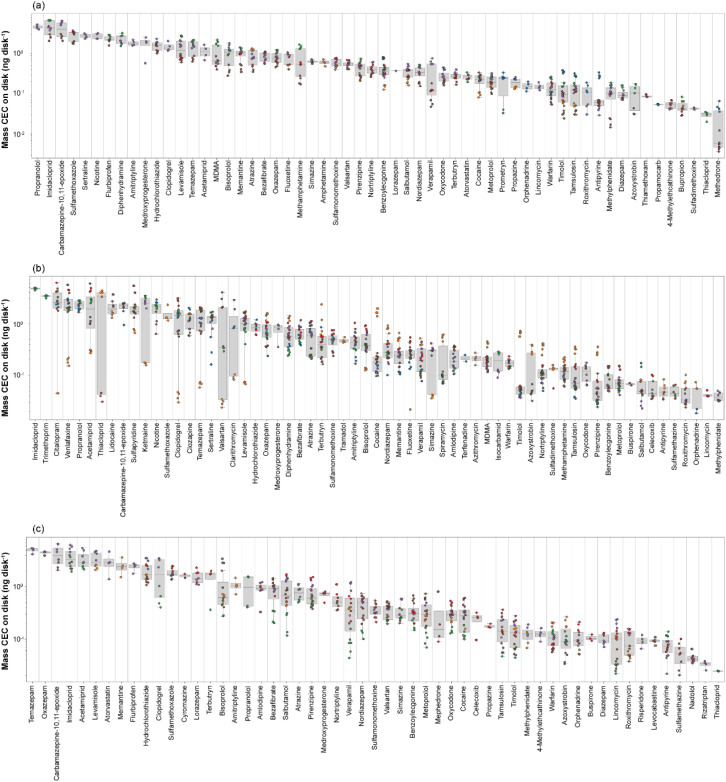
Boxplots representing the range of contaminant concentration in (a) HLB PSDs, (b) MM-anion PSDs, (c) MM-cation PSDs. Coloured dots indicate the month of sample collection, blue = July, orange = August, green = September, red = October, purple = November, and brown = December. Refer to S2 for monthly data.

**Fig. 3:**
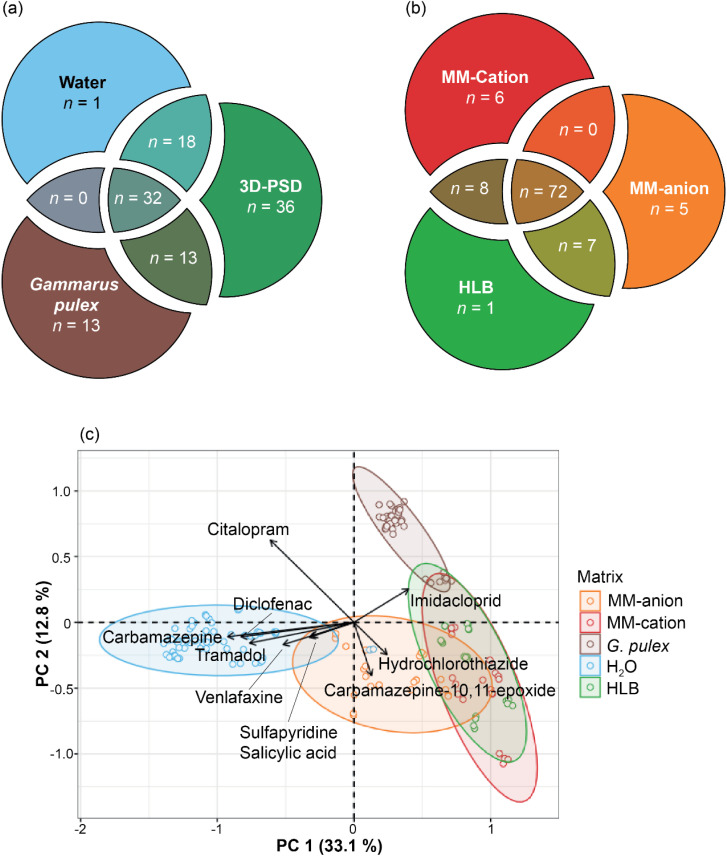
Venn diagrams representing the overlap in contaminant detections across the three matrices (a) and the different sorbent chemistries (b). Principal component analysis of all concentration data collected during the study per sample, loading vectors represent the top ten chemicals contributing to the spatial separation of samples (c). Axis are labelled with the respective principal component (PC) scores and the shaded ellipses represent a 95% confidence interval for each dataset. All data was scaled by contaminant within measurement samples from 0 to 1 to avoid bias.

None of the 3D-PSDs deployed during the study were lost or damaged. A total of 99 unique compounds were detected on the 60 passive samplers analysed and across all three sorbents. There was a negligible difference in the contaminant mass accumulated on the 3D-PSDs multiplexed with the three different sorbent chemistries and the devices containing only a single sorbent chemistry. This indicated that multiplexing different sorbents within the same 3D-PSD did not significantly affect CEC accumulation. Eighty CECs were quantifiable in at least one extract (Fig. 2(a)–(c)), and 39 quantifiable compounds were common to all three sorbents. Eleven of these (*i.e.*, acetamiprid, atrazine, azithromycin, azoxystrobin, clarithromycin, imidacloprid, sulfamethoxazole, terbutryn, thiacloprid, thiamethoxam, and trimethoprim) are currently/have been listed on the EU Water Framework Directive Watch Lists or are priority pollutants.^54^ The addition of the MM-anion and MM-cation sorbents extended the detectable chemical space captured by the HLB sorbent by eleven compounds (cymoxanil, cyromazine, dimethomorph, isocarbamid, ketotifen, levocabastine, picoxystrobin, pyraclostrobin, rizatriptan, sulfamerazine, and terfenadine) (Fig. 3(b)). However, the chemical space could potentially be further increased in future studies by adding other sorbent chemistries such as C_18_, silicone, or Tenax.^55^

In most cases, no significant contamination was observed in the field or extraction blanks throughout the study. In isolated instances where there was contamination, that specific compound was removed from that month's dataset. The CEC concentrations on disk across all sorbents ranged from 0.004 ng per disk (mephedrone, HLB phase in December 2021) to 6.7 ng per disk (imidacloprid, HLB phase in September 2021). The highest CEC concentrations were observed on the MM-cation phase (0.8 ± 1.0 ng per disk, median = 0.3 ng per disk), though more compounds were quantified on the MM-anion phase. There was no obvious or statistically significant monthly/seasonal trend in the CEC concentrations determined in 3D-PSDs across the full timeframe, including with respect to river flow. However, the highest average CEC mass on the sampler was noted in November for the HLB and MM-cation phases, and in October for the MM-anion phase (Fig. 2(a)–(c)). The highest concentration of any single compound over the whole study was imidacloprid (6.7 ± 0.1 ng per disk) in September 2021 on the HLB phase. Imidacloprid was also readily quantifiable in all water samples collected (mean = 66 ± 20 ng L^−1^), with an increased load in September 2021 (89 ± 20 ng L^−1^) matching trends in the HLB data (Fig. 2(a)).

A total of 58 CECs were quantifiable on the HLB phase, with November representing the highest cumulative mass on disk (48 ng per disk, 46 compounds) and August the lowest (4 ng per disk, 14 compounds). On the MM-anion disk, 63 compounds were quantifiable throughout the study, with the highest cumulative mass occurring in September (35 ng per disk, 42 compounds) and the lowest in November (15 ng per disk, 37 compounds). On the MM-cation disks, 55 compounds were quantifiable, with the highest total CEC mass occurring in November (58 ng per disk, 41 compounds, Fig. 2(c)) and the lowest in September (11 ng per disk, 25 compounds).

#### 3.1.3. CEC concentrations in *Gammarus pulex*

Fifty-eight compounds were detected in *G. pulex*. Of these, 26 were quantifiable and ranged from 3.8 ng per g (lidocaine) to 127.1 ng per g (citalopram), Fig. 1(b). On average, the most concentrated CEC class in *G. pulex* were insecticides (49.5 ± 26 ng g^−1^), followed by antidepressants (36.3 ± 39 ng g^−1^). Fourteen compounds were quantifiable in every *G. pulex* sample, including amlodipine, carbamazepine, clarithromycin, clopidogrel, clozapine, diphenhydramine, fluoxetine, imidacloprid, methamphetamine, propranolol, sulfamethoxazole, sulfapyridine, sulfathiazole, and venlafaxine. For many of these compounds, and particularly for substances affecting the human central nervous system, we showed that a rising prescription use in London led to increased riverine concentrations measured in river water across the city.^38,39^ This showed a direct impact of changing mental health on the water quality in these rivers, including in the urban areas through which the River Wandle runs. When considering the total CEC concentrations in biota measured here in the Wandle over the six months, there was a strong decreasing monthly trend in line with decreasing water temperatures moving from summer to winter. This was predominantly driven by citalopram, diphenhydramine, sulfapyridine, and warfarin (Fig. S2). These observations aligned with previous work that reported temperature-related changes in bioaccumulation and toxicity for aquatic invertebrate species for a variety of organic and inorganic contaminants.^56–59^

#### 3.1.4. Comparison of CEC occurrence across sample types

Compound occurrence in each of the three sample types (water, *G. pulex*, and the 3D-PSDs) is shown in Fig. 3(a) and (b). Thirty-two compounds were commonly detected across all three matrices, and 14 of these were quantifiable in all matrices. Thirteen of these were pharmaceuticals, including compounds that are named on the priority pollutant or the EU Water Framework Directive Watch Lists (Fig. 3(a) and Table S2). Imidacloprid was the only pesticide in common across all matrices. There were no compounds common to both the water and *G. pulex* samples alone (Fig. 3(a)). By comparison, there were 13 compounds commonly and uniquely detected in PSDs and *G. pulex* samples. Out of 50 compounds detected in water, all were also detected in at least one 3D-PSD sorbent chemistry. Despite the differences in CEC occurrence between the water and *G. pulex* samples, the selectivity of 3D-PSD sorbents was found to be broadly applicable across both matrices. Across all 3D-PSD sorbent chemistries, 49 additional compounds were detected on the 3D-PSDs that were either not detected or below the limit of quantification in the water samples (Table S2), and of these, 43 were quantifiable.

It is difficult to reliably compare contaminant concentrations derived from infrequent water grab samples to the TWA concentrations derived from 3D-PSDs using calibrated *R*_s_ values.^23^ Refer to SI S5 and Table S5 for details on *R*_s_ calibration experiments. Nonetheless, there was generally good agreement between the derived TWA CEC concentrations and those measured directly in the water grab samples collected during deployment (mean error = −18 ± 39 ng L^−1^, −19 ± 150 ng L^−1^, and 1 ± 41 ng L^−1^ for the HLB, MM-anion, and MM-cation phases, respectively, refer to Table S6 for a comparison of all compounds). For 11 compounds, the TWA CEC concentration in water could be determined for all sorbent phases and of those, only three (azoxystrobin, tamsulosin, and terbutryn) were in good agreement across phase chemistry (less than two standard deviations, Table S6). The discrepancy in TWA concentrations for the same compound across different sorbents is likely due to different and/or changing conditions in the river (*e.g.*, temperature, pH, water flow *etc.*), altering CEC interactions with each of the three sorbents.^28,60^

### 3.2 Environmental risk assessment and the estimation of toxic/effect pressures from contaminants

**Fig. 4:**
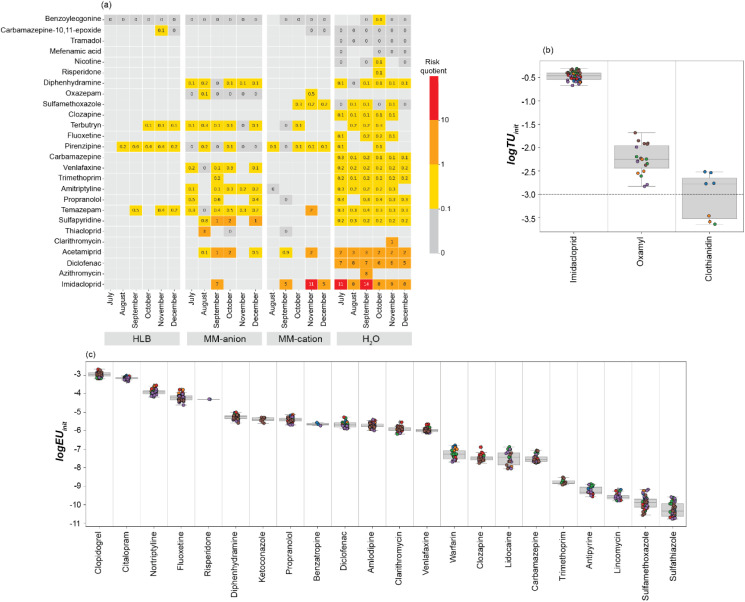
(a) Heatmap of the risk quotients (RQs) calculated for compounds with corresponding *R*_s_ values using the TWA concentration derived from the mass of contaminant accumulated on the HLB, MM-anion, MM-cation passive sampler sorbents, and from the contaminant concentration measured directly in water for compounds with a RQ > 0.1. Light grey tiles indicates compounds not quantified, compounds with a RQ of 0 are <0.1. Refer to Fig. S3 for all compounds. (b) Toxic units of pesticides quantified in *G. pulex* (the horizontal dotted line indicates a −3.0 threshold of risk). (c) Effect units of pharmaceuticals and personal care products quantified in *G. pulex*. Toxic and Effect units are log-transformed for ease of visualization. Coloured dots indicate the month of sample collection, blue = July, orange = August, green = September, red = October, purple = November, and brown = December.

Environmental risk quotients (RQs) were calculated for CECs accumulated on the 3D-PSDs for which there were *R*_s_ data (Table S5). The highest RQs were for imidacloprid on the MM-anion and MM-cation phases and pirenzepine on the HLB phase (Fig. 4(a) and S3). The RQs of imidacloprid were not determined on the HLB phase due to a lack of *R*_s_ data, but it was consistently above the medium risk threshold (*i.e.*, RQ > 1.0) for every month where there was quantifiable CEC occurrence data for water. The predicted no-effect concentration (PNEC) of imidacloprid is among the lowest used in this study (6.8 ng L^−1^).^42^ With respect to other acute (200 ng L^−1^) and chronic (35 ng L^−1^) limits proposed in the literature,^61^ all monthly MECs were above the chronic threshold for both water and 3D-PSD data, and none were above the acute value.^62,63^

To assess the risk of contaminants found in the *G. pulex* tissues, the internal toxic (log TU_int_, used for the assessment of pesticides) and effect (log EU_int_, used to assess pharmaceuticals) units were determined for each compound (Fig. 4(b) and (c)). The toxic units were calculated for three pesticide compounds (clothianidin, imidacloprid, and oxamyl). All toxic units for imidacloprid in *G. pulex* samples were greater than the −3.0 log TU threshold (based on concentrations measured in water) above which pesticides can have an adverse effect in invertebrates.^37,64,65^ With respect to London's rivers in particular, we found 21 compounds that posed a risk to freshwater aquatic life across 2019–2021.^38,39^ Of these, imidacloprid was also ranked as one of the highest risk compounds.

Imidacloprid is a neonicotinoid insecticide that was banned from outdoor use in the EU since 2018, but has since been identified in the environment, potentially from wastewater sources originating from widespread prophylactic use as a pet parasiticide to control ticks and fleas.^62^ Though this compound has a relatively short half-life, this consistent discharge source results in ‘pseudo-persistence’ in aquatic ecosystems.^63^ Imidacloprid is selectively toxic towards invertebrates, as it acts on the nicotine acetylcholine receptor in the central nervous system that is not present in vertebrates.^66^ It has been shown to adversely affect *G. pulex* mobility and feeding behaviour in 14-day exposures at 15 μg L^−1^.^67^ While the concentrations observed in the River Wandle are over 200-fold lower than this concentration, the effects of imidacloprid on wild aquatic invertebrates are still poorly understood and warrant further research given its continued prevalence.

Effect units were determined for the remaining 22 pharmaceutical compounds present in the *G. pulex*, aside from sulfapyridine, due to the lack of PC_crit_ data. Clopidogrel and citalopram presented the highest effect units (−3.1 ± 0.1 and −3.3 ± 0.1, respectively), likely driven by their very low PC_crit_ values: 6.4 × 10^−6^ mg L^−1^ and 1.4 × 10^−4^ mg L^−1^, respectively (Table S7). However, it is difficult to interpret the degree of risk caused by these compounds as the PC_crit_ values are not a toxicity endpoint^37^ and there are no established effect unit-based thresholds. Therefore, the estimation of effect units only serves to potentially rank compounds. In addition, caution should be taken around drawing conclusions from effect units as the PC_crit_ values used in the calculation were derived from HtPC data that may not necessarily induce an effect in the invertebrate. As the global detection of pharmaceuticals increases, there is a growing need to accurately quantify risk to species. In the absence of EC50 data for the vast majority of pharmaceutical compounds, PC_crit_ values can be used to estimate which compounds could elicit a pharmacological effect at a given tissue concentration.^37^ Therefore, this provided a means to preliminarily shortlist compounds for further investigation. Regardless, to improve the reliability of the approach, there is a need to generate pharmaceutical EC50 data for invertebrate taxa or further research into the read-across between the steady-state plasma concentration in humans, fish, and invertebrates is required.

### 3.3 Relating *in situ* environmental data to CEC tissue concentrations in *G. pulex*

Data collected from the six-month study was explored to determine if there were associations between the different matrices. Initially, any linear correlations between the *G. pulex* tissue concentrations and the CEC concentrations measured directly in water and the CEC mass on the 3D-PSD sorbents were investigated (Fig. S4). There was no significant relationship between the *G. pulex* and the MM-anion/water data (*p* > 0.05). There was a statistically significant relationship between the CEC concentrations found in *G. pulex* and both the HLB and MM-cation phases (*p* < 0.05). However, there was not a strong predictive correlation in either case (*R*^2^ = 0.307 and 0.461, respectively, Fig. S4). These findings align with previous work by Grabicová *et al.* (2022), who determined that there was a significant but low predictive relationship (*R*^2^ = 0.485, *p* < 0.05) between total pharmaceutical concentrations in benthic invertebrates and total uptake onto a HLB-POCIS device.^68^ This indicated that environmental water concentrations measured using grab samples and passive samplers are not a good ‘proxy’ for *G. pulex* tissue concentrations.

Principal component analysis was then used to visualise and assess the chemical variance across the datasets for all samples. Each matrix formed distinct clusters with minimal overlap, except for the 3D-PSD sorbents, likely due to the shared PS-DVB base polymer between the different sorbents (Fig. 3(c)). However, the 3D-PSD data was spatially closer to the *G. pulex* data than the water, primarily driven by imidacloprid. This indicated that while none of the matrices were effective at characterising chemical occurrence, the variance explained in the *G. pulex* data were more aligned with that of the 3D-PSD data than the river water samples. Hierarchical clustering analysis (Fig. S5) supported this association, with the *G. pulex* extract samples clustered with 3D-PSD sorbents. This potentially indicated a shared uptake mechanism between the 3D-PSD and *G. pulex*. There is preliminary evidence supporting this when correlating the 3D-PSD uptake rate (*R*_s_) and contaminant concentration in the *G. pulex* for the MM-anion sorbent phase (Fig. S6), and a similar relationship has been observed between other passive sampler devices and invertebrates.^69,70^

### 3.4 Correlating toxic and effect units in *G. pulex* and 3D-PSDs

As the direct linear relationship between analyte concentration in the *G. pulex* and the environmental water samples did not reveal an adequate predictive relationship (*R*^2^ < 0.7), a novel alternative approach was tested. In effect, by mathematically treating the 3D-PSD as an organism in itself, it is possible to calculate the toxic/effect units for the 3D-PSD. This was performed by substituting the mass of contaminant on the 3D-PSD (adjusted for the weight of the disk = 0.0115 g) for the *C*_invert_ term in eqn (2) and (4). Importantly, this potentially removes the need to use calibrated *R*_s_ data to determine and prioritise CEC risk in the *G. pulex*, as it can be done directly from the compounds quantified on the 3D-PSDs.

#### 3.4.1. Toxic units

Imidacloprid was the only compound for which EC50 data existed for *G. pulex* and this substance was present in both the 3D-PSD and *G. pulex*, so the other two pesticides were not considered further. log_10_ TU_int_ across all sorbents lay above the −3.0 threshold (HLB = 0.3 ± 0.2 log_10_ units, MM-anion = 0.3 ± 0.1 log_10_ units, MM-cation = 0.2 ± 0.2 log_10_ units) and more similar to the values calculated in *G. pulex* (−0.5 ± 0.1 log_10_ units) than those calculated from the water data (−2.5 ± 0.1 log_10_ units) as per eqn (2) using unadjusted EC50 values.^71,72^ This indicated that while none of the matrices were effective at characterising chemical occurrence, calculations using the 3D-PSD data may hold promise for estimation of *G. pulex* risk over water-based assessments and as initially indicated by PCA (Fig. 3(c)).

This approach was further evaluated using data mined from literature studies where water and *Gammarid* invertebrates were co-analysed.^15,37,43,73^ Across the studies, four pesticides (azoxystrobin, imidacloprid, propamocarb, and thiacloprid) were quantified in the *Gammarid* and water samples from a range of freshwater and estuarine systems. Concentrations of CECs in water and *Gammarid* replicates for each compound per site within each study were averaged. In this case, contaminant mass on the 9 mm sorbent disk was instead calculated from the literature water data using known *R*_s_ values (Table S5 & Richardson *et al.*, 2022), and the toxic units were calculated as above. Across all studies, imidacloprid was the only pesticide to exceed the −3.0-effect threshold calculated from concentrations in the *Gammarid* tissue (Table S8). However, toxic units calculated from the water concentration data from each of the corresponding sites did not exceed this threshold, thereby incorrectly classifying the risk. Using the theoretical mass on 3D-PSD for the MM-anion and MM-cation phases (HLB toxic units for imidacloprid could not be calculated due to lack of *R*_s_ values), correctly classified the threshold exceedance. However, the toxic units calculated using the 3D-PSD incorrectly classified the risk for azoxystrobin compared to that measured directly in the *Gammarid* (Table S8). This could be due to an error in the assumption that the uptake rates for azoxystrobin calculated in-laboratory for the 3D-PSD are consistent across water systems, which is not always the case.^60,74^ Multi-site *in situ* calibration studies could be used to assess the applicability of sampling rates across different environments.^75^ Further laboratory-based testing should be performed to further verify the relationships observed here for more compounds, where toxic units can be calculated reliably in *G. pulex*. However, as a proof-of-concept application in the field, this still represented a very promising result.

#### 3.4.2. Effect units

For all remaining compounds, and when modelled separately, the linear comparison between effect units calculated in the *G. pulex* and the 3D-PSD sorbents resulted in a statistically significant (*p* < 0.05, *R*^2^ > 0.86) correlation (Fig. S7(a)–(c)). The mean error of the different sorbent models for calculating the effect units in the *G. pulex* was 3.3 × 10^−16^ ± 0.6 (95% CI [−0.3, 0.3]), −0.1 ± 0.5 (95% CI [−0.2, 0.1]), and −6.5 × 10^−4^ ± 0.5 (95% CI [−0.2, 0.2])log_10_ units for the HLB, MM-anion, and MM-cation models, respectively (Fig. S7(d)–(f)). Because the contaminant concentration in the invertebrate is a term (*C*_invert_, eqn (4)) used to calculate the effect unit risk, when this is known, the concentration in the invertebrate can be calculated. Using this method, the mean error of the internal *G. pulex* concentrations modelled using this new approach were −11 ± 38 (95% CI [−29, 6]), −7 ± 35 (95% CI [−16, 3]), and −9 ± 28 (95% CI [−22, 4]) ng g^−1^, for the HLB, MM-anion, and MM-cation sorbent phases, respectively (Fig. S7(g)–(i)). All three models were validated using k-fold cross-validation; due to the limited size of the datasets (*n* = 20 to 52), only five folds were used. The average mean errors of calculating the *G. pulex* effect units for each model across all validation runs were −0.01 ± 0.2 (95% CI [−0.4, 0.5]), 0.05 ± 0.3 (95% CI [−0.4, 0.5]), and 0.05 ± 0.3 (95% CI [−0.3, 0.4])log_10_ units for the HLB, MM-anion, and MM-cation models, respectively. The mean errors of calculating the internal *G. pulex* concentrations were −10 ± 12 (95% CI [−39, 14]), −7 ± 19 (95% CI [−30, 17]), and −12 ± 21 (95% CI [−24, 5]) ng g^−1^ for the HLB, MM-anion, and MM-cation models, respectively.

The underlying data was interrogated to identify and understand the strong correlation between the effect units calculated from 3D-PSD and *G. pulex* data. When comparing the log-transformed normalised mass on sampler and log-transformed concentration in *G. pulex*, there was not a strong linear relationship (*R*^2^ < 0.4) across any of the sorbent chemistries. Therefore, the denominator of the effect unit calculations (PC_crit_) had a strong influence on the correlation, despite being common across both axes. An important consideration, however, is that deployed 3D-PSDs are static, and movement of *G. pulex* was not accounted for in this *in situ* experiment. As stated above, there is uncertainty in the validity of the application of effect units to invertebrates and the implications for risk. However, the strength of the relationship between the effect units calculated in the 3D-PSD and *G. pulex* presented here warrants further investigation to determine if the relationship is conserved when using PC_crit_ values calculated in invertebrates.

## 4. Conclusion

This work represents, for the first time, the evaluation of a multi-modal 3D-PSD to estimate the risk and internal CEC concentration in a freshwater invertebrate while accounting for *in situ* environmental conditions. Over a six-month temporal study of the River Wandle where water, *G. pulex*, and passive samplers containing different sorbent chemistries (HLB, MM-anion, and MM-cation exchange), 112 unique CECs were identified (water = 50; 3D-PSDs = 99; and *G. pulex* = 58 CECs). A total of 80 CECs were quantifiable on the 3D-PSD sorbents (HLB = 58, MM-anion = 63, and MM-cation = 55) and 41 in water. A total of 26 CECs were quantified in the *G. pulex* samples, of which 19 were common to those found in 3D-PSDs (16 common to water). Imidacloprid was the most concentrated compound in 3D-PSDs (6.8 ng per disk), was calculated to have a medium to high environmental risk (RQ > 1 using water and MM-anion/MM-cation passive sampler data) and was calculated to pose the highest toxic unit for *G. pulex*. Principal component analysis indicated that there was a small degree of overlap between the 3D-PSD and *G. pulex* data, primarily driven by imidacloprid. The mass accumulated on 3D-PSD sorbents was also used to estimate toxic and effect units in *G. pulex* in separate models. However, the interpretation of the risk posed by pharmaceuticals, in particular, using these models remains challenging in the absence of toxicological data specific to *G. pulex*. That said, we recommend the current 3D-PSD design to reduce the reliance on invertebrate testing in the field as it can aid with prioritisation of monitoring sites or bioavailable CECs to study. Future work combining the 3D-PSD and machine learning tools that model and eventually predict toxicological information for invertebrates and across species would be of great benefit and aid in regulatory prioritisation of emerging contaminants in a policy framework. However, this model cannot yet be used as a replacement for biota, as it does not capture the wider biological impacts (*e.g.*, genetic or behavioural changes arising from exposure). Therefore, it is recommended that future work focus on widening the number and range of CECs to improve the model's generalisability, as well as an evaluation of performance in different waterbodies and for application to other aquatic organisms. As a successful proof-of-concept study, however, this represents an advancement in the use of PSDs for invertebrate risk assessment for CECs and is easily deployable at large scales.

## Author contributions

Alexandra K. Richardson: conceptualisation, methodology, validation, formal analysis, resources, software, investigation, data curation, visualisation, writing – original draft, writing – review & editing. Stephen Stürzenbaum: supervision, writing – review & editing. David A. Cowan: supervision, writing – review & editing. David J. Neep: resources, writing – review & editing, funding acquisition, supervision. Leon P. Barron: conceptualisation, methodology, resources, writing – review & editing, supervision, project administration, funding acquisition.

## Conflicts of interest

The authors declare no conflict of interest.

## Supplementary Material

EM-028-D5EM00452G-s001

EM-028-D5EM00452G-s002

## Data Availability

The data supporting this article have been included as part of the supplementary information (SI). Supplementary information: it describes the list of reference materials; additional sampling and sample preparation details information on individual CEC occurrence across matrices, time and temperature ranges; method performance metrics; 3D-PSD calibration approach and *R*_s_ data; and statistical and correlative analysis of occurrence data across each matrix for risk modelling using 3D-PSDs. See DOI: https://doi.org/10.1039/d5em00452g.
