# Supplementary material for: Multimodal 3D-printed passive samplers to monitor, model and prioritise in situ pharmaceutical and pesticide pollution risks to an aquatic freshwater invertebrate, Gammarus pulex
Source: Environ Sci Process Impacts. 2025 Oct 14;28(1):42–55. doi: 10.1039/d5em00452g (PMC12536334; doi:10.1039/d5em00452g)
Supplement: EM-028-D5EM00452G-s002 [file EM-028-D5EM00452G-s002.pdf]

Supplementary Information (SI) for Environmental Science: Processes & Impacts.
This journal is © The Royal Society of Chemistry 2025

Supplementary Information

Multimodal 3D-printed passive samplers to monitor, model and prioritise in situ chemical pollution risks to an aquatic freshwater invertebrate, Gammarus pulex

Alexandra K Richardson[1,2], Stephen Stürzenbaum[2], David A Cowan[2], David J Neep[3] and Leon P Barron[1, 2*]

[1] *MRC Centre for Environment & Health, Environmental Research Group, School of Public Health, Faculty of Medicine, Imperial College London, 86 Wood Lane, London W12 0BZ, United Kingdom.*
[2] *Dept. Analytical, Environmental & Forensic Sciences, Institute of Pharmaceutical Sciences, School of Cancer and Pharmaceutical Sciences, Faculty of Life Sciences & Medicine, King's College London, 150 Stamford Street, London, SE1 9NH, United Kingdom.*
[3] *Agilent Technologies UK Ltd, Essex Road, Church Stretton, SY6 6AX, United Kingdom.*

* Corresponding author: leon.barron@imperial.ac.uk

S1. List of analytical standards

Reference standards for 2-(thiocyanomethylthio)benzothiazole, 4-fluoromethacationone, 4-methylethcathinone, acetamiprid, aclonifen, alprazolam, ametryn, amiodarone, amitriptyline, amlodipine, amphetamine, antipyrine, atorvastatin, atrazine, azelnidipine, azithromycin, azoxystrobin, benoxacor, bensulide, benzatropine, benzoylecgonine, betaxolol, bezafibrate, bisoprolol, bupropion, buspirone, carazolol, carbamazepine, carbamazepine-10, 11-epoxide, carboxine, carfentrazone-ethyl, celecoxib, chloramphenicol, cilazapril, citalopram, clarithromycin, clodinafop-propargyl, clofibric acid, clopidogrel, clothianidin, clozapine, cocaine, cyclouron, cycloxyidim, cymoxanil, cyphenothrin, cyromazine, diazepam, diclofenac, diflubenzuron, dimethametryn, dimethomorph, diphenhydramine, disulfoton sulfone, diuron, enalapril, ethofumesate, famoxadone, fenoxaprop-ethyl, fenuron, flufenoxuron, fluocinonide, fluoxetine, flurbiprofen, flurochloridone, flutamide, flutolanil, fuberidazole, gemfibrozil, haloperidol, hydrochlorothiazide, ibuprofen, imidacloprid, indomethacin, isocarbamid, isradipine, josamycin, ketamine, ketoconazole, ketoprofen, ketotifen, levamisole, levocabastine, lidocaine, lincomycin, lorazepam, MDMA, meclizine, meclofenamic acid, medroxyprogesterone, mefenamic acid, memantine, mephedrone, mephosfolan, metformin, methamphetamine, methcathinone, methedrone, methylphenidate, metoprolol, morphine, nadolol, naproxen, nicotine, nifedipine, nitenpyram, nordiazepam, norethisterone, nortriptyline, orphenadrine, oxamyl, oxazepam, oxycarboxin, oxycodone, picoxystrobin, piperophos, pirenzipine, pretilachlor, prodiamine, prometon, prometryn, propamocarb, propranolol, propazine, pymetrozine, pyracarbolid, pyraclostrobin, pyraflufen-ethyl, pyridaben, risperidone, rizatriptan, ronidazole, roxithromycin, salbutamol, salicylic acid, sertraline, simazine, spinosyn A, spinosyn B, spiramycin, sulfadimethoxine, sulfamerazine, sulfamethazine, sulfamethoxazole, sulfamonomethoxine, sulfapyridine, sulfathiazole, sulfisoxazole, tacrine, tamsulosin, temazepam, terbutryn, terfenadine, thiacloprid, thiamethoxam, thiazopyr, timolol, tramadol, trimethoprim, valsartan, venlafaxine, verapamil, warfarin and ziprasidone were sourced from QMX (Essex, UK).

Deuterated internal standards for amitriptyline-d3, amphetamine-d6, benzoylecgonine-d3, betaxolol-d7, celecoxib-d7, cetrizine-d4, clarithromycin-d3, clothianidin-d3, cocaine-d3, cotinine-d3, diazepam-d6, fluoxetine-d6, gemfibrozil-d6, haloperidol-d4, ketamine-d4, lidocaine-d10, lorazepam-d4, MDMA-d5, methylone-d3, methylphenidate-d9, metoprolol-d7, morphine-d3, nicotine-d4, nifedipine-d4, nordiazempam-d5, nortriptyline-d3, oxazepam-d5, risperidone-d4, sertraline-d3, sulfamethazine-d4, temazepam-d5, thiamethoxam-d3, tramadol-d3, trimethoprim-d3, venlafaxine-d6 and verapamil-d3 were purchased from Sigma Aldrich (Gillingham, Dorset, UK) and QMX (Essex, UK).

Table S1. Collection events per month across the course of the study for all sample types

**Month & collection event**		**Water**	**3D-PSD**	***Gammarus pulex***
July	Deployment – 20th	✓		✓
	Midpoint – 24th	✓		✓
	Retrieval – 27th	✓	✓	✓
August	Deployment – 10th	✓		✓
	Midpoint – 15th	✓		✓
	Retrieval – 17th	✓	✓	✓
	Extra – 27th	✓		✓
September	Deployment – 7th	✓		✓
	Midpoint – 10th	✓		✓
	Retrieval – 14th	✓	✓	✓
	Extra – 27th	✓		✓
October	Deployment – 13th	✓		✓
	Retrieval – 20th	✓	✓	✓
	Extra – 29th	✓		✓
November	Deployment – 9th	✓		✓
	Midpoint – 12th	✓		✓
	Retrieval – 16th	✓	✓	✓
	Extra – 26th	✓		✓
December	Deployment – 7th	✓		✓
	Midpoint – 12th	✓		✓
	Retrieval – 14th	✓	✓	✓

S2. Site selection and sample collection - additional details

Of the ten 3D-PSDs deployed each month, three devices contained the same chemistry for all sorbent disks contained within the same device (i.e., one 3D-PSD contained five HLB sorbent disks) for the HLB, MM-anion, and MM-cation phases, respectively. In addition, a single device was multiplexed with all three sorbent chemistries and deployed alongside the other 3D-PSDs. The 3D-PSDs were securely anchored to the riverbed as described in Richardson *et al.* (2022) using U-shaped galvanised pegs (15 x 0.29 cm, G&B, UK) which were pushed into the sediment so that the 3D-PSD was sitting approximately 3-5 cm from the bottom of the river with the water flowing over the top of the device (Richardson et al., 2022). During retrieval, the 3D-PSDs were removed from the riverbed and rinsed in river water before the transport cap was fitted, the device was then wrapped in methanol-washed aluminium foil and stored in a food-grade plastic container for transport to the laboratory in a cool box as standard practice in passive sampling (Taylor et al., 2021). A separate 3D-PSD was used as the field blank during deployment and retrieval to account for contamination occurring from handling the 3D-PSDs in the field and laboratory. Upon return to the laboratory, all 3D-PSDs were rinsed with ultra-pure water, disassembled, the PES membrane removed, and the sorbent disks were allowed to air dry overnight on MeOH rinsed foil alongside the field blanks. Once dry, the sorbent disks were wrapped in fresh MeOH-washed foil and stored at -20 °C until extraction and analysis.

G. pulex samples were collected by kick-sampling multiple organisms using a D-frame net (NHBS, Totnes, UK). The net composed of a stainless-steel frame with a wooden handle fitted with a woven polyester net (diameter 30 cm, depth 50 cm, 500 µm mesh size). Kick sampling is a sample collection technique where a net is placed downstream of the operator, and then the riverbed is disturbed by kicking the upper layers of sediment in a perpendicular fashion to the direction of flow to collect benthic organisms (Storey et al., 1991). An area of approximately 2-3 m^2 was covered to collect organisms and water depth at this site was relatively shallow (0.5 m at a maximum). All collected macroinvertebrates were returned to the laboratory and sorted using 1,000 and 500 µm sieves, where all *G. pulex* individuals larger than 0.5 mm were collected using forceps (n > 15 specimens). Pure *G. pulex* samples were stored at -20 °C in preparation for sample analysis and the other invertebrates (including polychaetes and larvae) and isopods (including *Asellus aquaticus*) were disposed of.

S3. Sample preparation - additional details

Extractions were performed using pre-fritted, empty SPE cartridges (Agilent Technologies UK Ltd., Cheshire, UK) configured to an SPE manifold with the taps closed. Before extraction, all sorbent disks were directly spiked with 0.5 ng of SIL-IS in MeOH and left to dry. Initially, 1 mL of elution solvent was added on top of the 9 mm disks and allowed to soak for 15 min. After which, the taps were opened and the elution solvent was allowed to percolate into 2 mL microcentrifuge tubes. An additional 0.6 mL of elution solvent was added to the SPE cartridges and gently pulled through under vacuum. The eluent was then filtered through 0.2 µm polytetrafluoroethylene (PTFE) membrane using a BD Plastipak^{TM} syringe (FisherScientific UK Ltd., Loughborough, UK) directly into pointed glass tubes (Merck Life Science UK, Ltd., Dorset, UK) for the subsequent evaporation step. Extracts were dried at 35 °C under N_2 before reconstitution in 200 µL of starting mobile phase conditions. Per month, n = 3 separate disks from one 3D-PSD device were extracted as measurement replicates. To perform quantification, the spare 9 mm disks from the same deployment were directly spiked with analytical standards to form an 11-point calibration series (0.005 to 10 ng disk^{-1}) per sorbent phase and extracted as above.

All frozen *G. pulex* samples at each timepoint (typically 15 to 50 individuals) were lyophilised at -40 °C under vacuum for at least 24 h. These were pooled and homogenised to a fine powder in a 2 mL microcentrifuge tube with a 3 mm tungsten carbide bead using a TissueLyser LT benchtop system (50 Hz, Qiagen, Hilden, Germany). Homogenised material was weighed into a new 2 mL microcentrifuge tube (20 ± 0.1 mg, n = 3 per collection timepoint), all analytical standards and SIL-IS (50 ng g^{-1}) were spiked directly onto the dried material in a total volume of 100 µL of MeOH. Samples were extracted in 2 mL of 0.1 % (*v/v*) glacial acetic acid in 3:1 MeCN:H_2O using an ultrasonic bath for 15 min. Insoluble material was pelleted out by centrifugation at 18,407 rcf for 5 min, 1.9 mL of the supernatant was removed and diluted in 100 mL of 10 mM ammonium acetate$_{(aq)}$. Sample clean-up and preconcentration was performed using tandem sold-phase extraction with an upper matrix removal cartridge (Strata Alumina-N cartridge, 6 mL, 1 g, Phenomenex Ltd., Cheshire, UK) and a lower Oasis HLB cartridge (6 mL, 200 mg, Waters Corp., Hertfordshire, UK) for analyte preconcentration (Miller et al., 2019). Cartridges were conditioned with 6 mL each of MeOH and 10 mM ammonium acetate$_{(aq)}$ prior to sample loading. The SPE system was subsequently washed with 1 mL of ultra-pure water, the upper cartridge removed, and the lower cartridge dried under vacuum. Analytes were eluted with a total of 5 mL of MeOH split into two 2.5 mL aliquots and dried under N_2 at 35 °C before reconstitution in 100 µL of initial mobile phase conditions, briefly centrifuged twice for 5 min at 254 rcf to pellet any particulates that formed during the reconstitution step before transfer to silanised HPLC vial inserts for analysis. Quantification was performed by comparing to an 11-point calibration series (0.05 to 150 ng g^{-1}) using homogenised *G. pulex* material collected from a relatively contaminant-free site in the south of London (51.385972; 0.109000, Miller et al., 2017, 2015) and prepared as above.

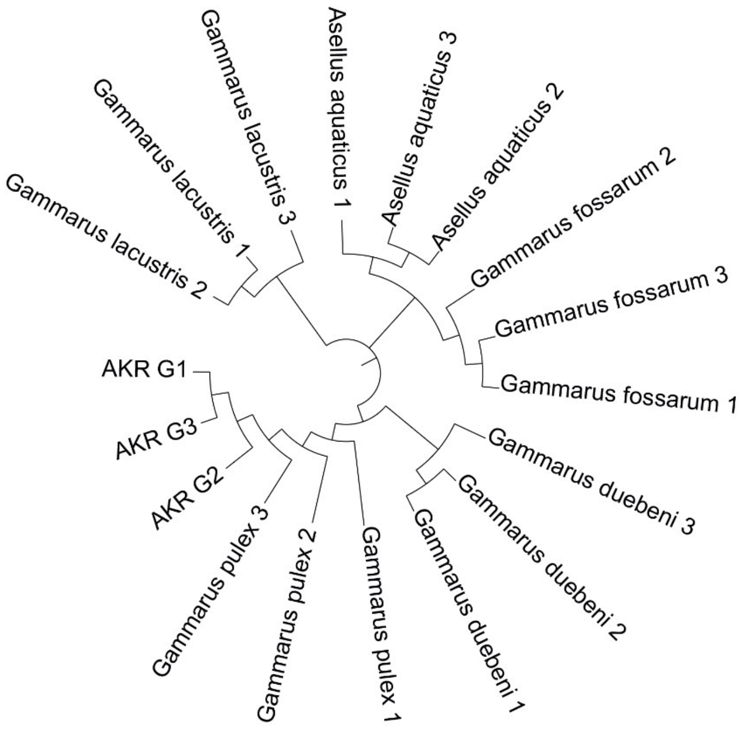

Figure S1. *Maximum likelihood estimation phylogenetic tree generated from Cytochrome C Oxidase (COI) DNA barcode sequencing comparing three individuals (AKR G1-3) collected from the Gammarid population at the Ravensbury study site to published known COI sequences from four freshwater gammarid species (G. pulex, G. duebeni, G. lacustris, and G. fossarum) and an evolutionary distant water louse (Asellus aquaticus). Forward (5'-ACGTCATAGTCACCGCTCAC-3') and reverse (5'-CGTTCACCCTGTTCCTACCC-3') primers were designed using the Primer3Plus platform (Untergasser et al., 2012). Reference COI sequences (S4) were retrieved from the National Center for Biotechnology Information (NCBI) GenBank (Benson et al., 2013). Results indicate that the sampled individuals share a common ancestor with G. pulex. Created using MegaX software and maximumhood likelihood estimation was used.*

S4. Aligned COI haplotype sequences with NCBI GenBank reference codes

G1
TATAATAATTGGTGGTTTCGGGAATTGACTGGTGCCATTAATGCTAGGTAGACC
TGATATAGCTTTTCCGCGTATAAACAATATAAGATTTTGACTTTTACCTCCTTCTC
TCACCCTTCTGCTTATAAGTAGTATAGTAGAAAGAGGGGTAGGAACAGGGTGAA
CG

G2
TTATAATTGGGTGGGTTCGGGGAATTGACTGGTGCCACTAATGCTAGGTAGACC
TGATATAGCTTTTCCGCGTATAAACAATATAAGATTTTGACTTTTACCTCCTTCTC
TCACCCTTCTGCTTATAAGTAGTATAGTAGAAAGAGGGGTAGGAACAGGGTGAA
GGA

G3
TATNATAATTGGTGGTTTCGGGAATTGACTGGTGCCATTAATGCTAGGTAGACC
TGATATAGCTTTTCCGCGTATAAACAATATAAGATTTTGACTTTTACCTCCTTCTC
TCACCCTTCTGCTTATAAGTAGTATAGTAGAAAGAGGGGTAGGAACAGGGTGAA
CG

Gammarus pulex1 (MW459741.1)
GATTATAATCGGGGGTTTCGGAAATTGACTAGTACCTTTAATATTAGGTAGACCT
GATATAGCTTTCCCGCGAATAAACAATATAAGATTTTGACTTTTACCCCCTTCCC
TAACACTTCTGCTTATAAGTAGTATAGTAGAAAGAGGGGTAGGAACAGGGTGAA
CG

Gammarus pulex2 (MG986849.1)
TATTATAATTGGCGGTTTCGGAAATTGATTGGTTCCATTAATATTAGGTAGACCT
GATATAGCTTTCCCGCGTATAAATAATATAAGGTTTTGACTTTTACCTCCTTCTCT
AACCCTTCTACTTATAAGTAGTATAGTAGAAADAGGCGTAGGAACGGGTTGGAC
G

Gammarus pulex3 (KY464959.1)
TATTATAATTGGTGGTTTCGGGAATTGACTGGTGCCATTAATGCTAGGTAGACC
TGATATAGCTTTTCCGCGTATAAACAATATAAGATTTTGACTTTTACCTCCTTCTC
TCACCCTTCTGCTTATAAGTAGTATAGTAGAAAGAGGTGTAGGAACGGGTTGAA
CG

Gammarus duebeni1 (HF930488.1)
TATTATAATCGGTGGATTCGGTAACTGACTAGTGCCGCTTATGCTAGGTAGCCC
AGACATAGCCTTCCCGCGTATAAATAATATAAGATTTTGACTTTTACCCCCTTCT
CTAACTTTACTACTTATAAGGGGGCTTGTAGAAAGAGGGGTTGGTACAGGTTGA
ACC

Gammarus duebeni2 (KT209188.1)
TATTATAATCGGTGGATTCGGTAACTGACTAGTACCGCTTATGCTAGGTAGCCC
AGACATAGCCTTCCCCCGTATAAATAATATAAGATTTTGACTTTTACCCCCTTCT
CTAACTTTACTACTTATAAGGGGGCTTGTAGAAAGAGGGGTTGGTACAGGTTGA
ACC

Gammarus duebeni3 (HF930481.1)
TATTATAATCGGGGGATTCGGTAATTGACTAGTGCCGCTTATGCTAGGTAGCCC
AGACATAGCCTTCCCCCGTATAAACAACATAAGATTTTGACTTTTACCACCTTCT
CTAACTTTACTACTTATAAGGGGGCTTGTAGAAAGAGGGGTTGGCACAGGTTGA
ACC

Gammarus lacustris1 (KU376166.1)
TATCATAATTGGGGGGTTCGGCAACTGACTAGTGCCTTTAATACTAGGTAGACC
TGATATAGCTTTCCCTCGTATAAACAACATGAGATTCTGGCTTTTACCCCCCTCC
TTAACTCTTCTTCTTATAAGAGGTCTAGTGGANAGCGNAGTGNGTACTGGGTGA
ACT

Gammarus lacustris2 (FJ756329.1)
TATCATAATTGGGGGGTTCGGGAACTGACTAGTGCCTTTAATACTAGGTAGACC
TGATATAGCTTTCCCTCGTATAAACAACATGAGATTCTGGCTTTTACCCCCCTCC
TTAACTCTTCTTCTTATAAGAGGTCTAGTGGAGAGCGGAGTGGGTACTGGGTGA
ACT

Gammarus lacustris3 (DQ889100.1)
TATCATAATTGGAGGGTTCGGCAACTGACTAGTGCCTTTAATACTAGGTAGACC
TGATATAGCTTTCCCTCGTATGAATAACATGAGATTCTGGCTTTTACCCCCCTCC
TTAACTCTTCTTCTTATAAGAGGTCTAGTGGAGAGCGGAGTGGGTACTGGATGA
ACT

Gammarus fossarum1 (KY464960.1)
CATTATAATCGGCGGATTTGGAAACTGATTAGTTCCTTTAATGCTAGGTAGCCCT
GATATAGCCTTTCCACGTATAAATAACATAAGATTTTGATTATTACCCCCCTCATT
AACCCTGCTTCTAATAAGAAGTTTAGTAGAAAGGGGAGTGGGCACGGGTTGAA
CT

Gammarus fossarum2 (KX065397.1)
CATTATAATCGGCGGATTTGGAAACTGATTAGTTCCTTTAATGCTAGGTAGCCCT
GATATAGCCTTTCCACGTATAAATAACATAAGATTCTGATTATTACCCCCCTCAT
TAACCCTACTTCTAACAAGAAGTTTAGTAGAAAGGGGAGTGGGCACGGGTTGAA
CT

Gammarus fossarum3 (KY464962.1)
CATTATAATCGGCGGATTTGGAAACTGATTAGTTCCTTTAATGCTAGGTAGCCCT
GATATAGCCTTTCCACGTATAAATAACATAAGATTTTGATTACTACCCCCCTCAT
TAACCCTGCTTCTAATAAGAAGTTTAGTAGAAAGGGGAGTGGGCACGGGTTGAA
CT

Asellus aquaticus1 (AY531819.1)
AGTAATAATTGGAGGGTTTGGAAATTGATTAATTCCTTTAATACTAGGCGCCCCC
GATATAGCATTCCCTCGTATAAATAATATAAGATTCTGATTATTACCTCCCTCTCT
TACTCTACTCCTATCAAGAGGGTTAATTGAAAGAGGGGTAGGGACCGGTTGAAC
A

Asellus aquaticus2 (DQ144891.1)
AGTGATAATCGGAGGGTTTGGAAATTGATTAATTCCCCTAATGCTAGGTGCCCC
AGATATAGCATTTCCTCGCATAAATAATATAAGATTCTGACTATTACCTCCTTCTC
TAACCCTGCTCCTGTCTAGGGGGTTAATTGAAAGAGGAGTAGGAACTGGCTGA
ACA

Asellus aquaticus3 (MG986872.1)
AGTGATAATCCGACGGTTTGGAAATTGATTAATTCCCCTAATGCTAGGTGCTCC
AGATATAGCATTTCCTCGTATAAATAATATAAGATTCTGACTATTACCTCCTTCTC
TAACCCTGCTCCTGTCTAGGGGGCTAATTGAAAGAGGAGTAGGAACTGGCTGA
ACA

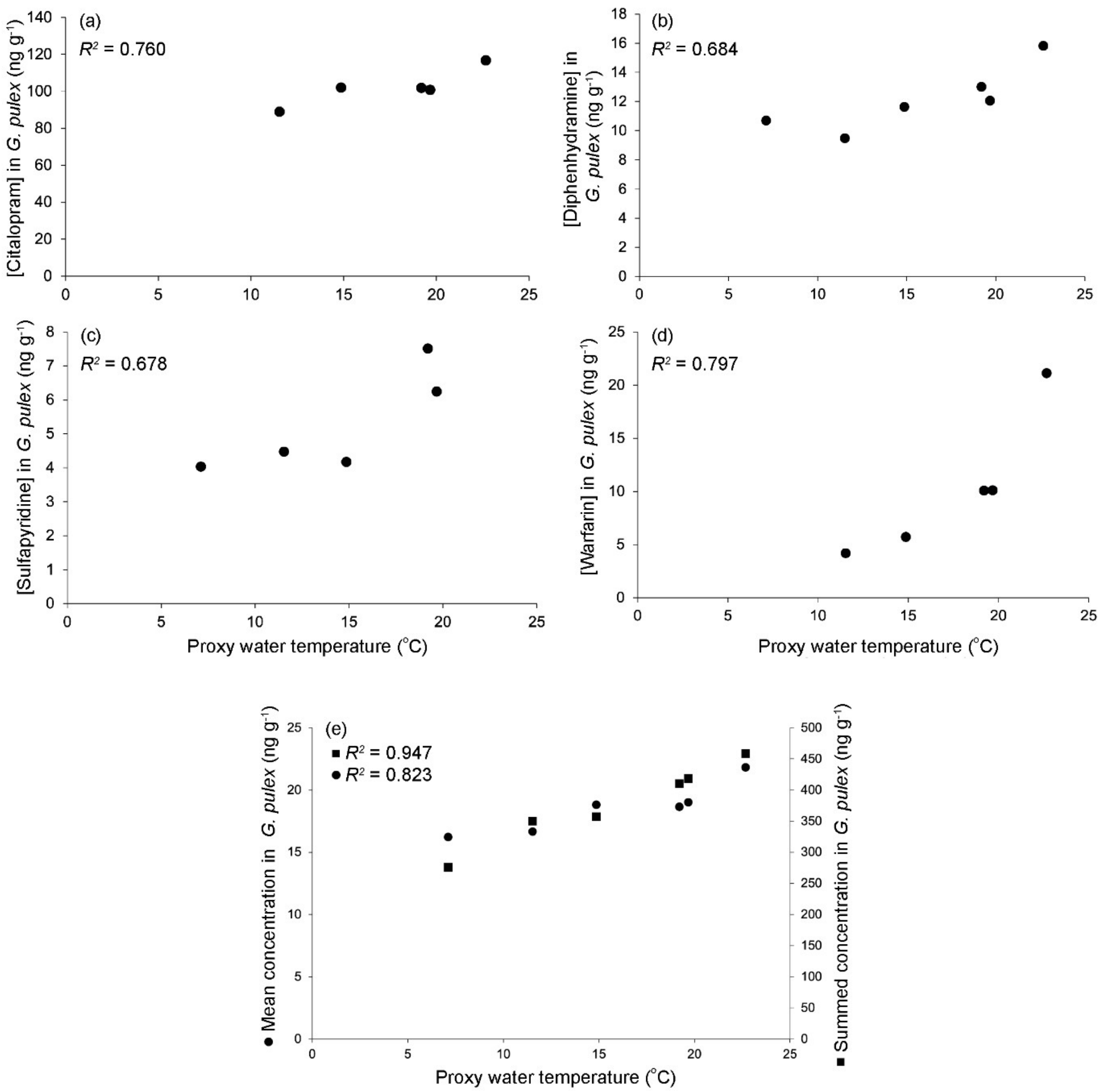

***Figure S2**. Linear relationships between the water temperature and the concentration of citalopram (a), diphenhydramine (b), sulfapyridine (c), and warfarin (d). R^2 values are indicated on the graphs. (e) Correlation between the mean concentration in G. pulex (left axis), cumulative concentration in the G. pulex (right axis) and proxy water temperatures.*

Table S2. Average concentration ± standard deviation of 89 compounds quantified over the entire 6-month study for each compound. Please refer to Tables S9 to S13 in the separate

supplementary data file for monthly data for all matrices.

**Compounds**	**HLB (ng disk-1)**	**MM-anion (ng disk-1)**	**MM-cation (ng disk-1)**	**G. pulex (ng g-1)**	**H_2O (ng L-1)**
4-Methylethcathinone	0.05 ± 0.01	< LLOQ	0.13 ± 0.03	-	-
Acetamiprid	1.15 ± 0.40	2.24 ± 1.80	3.39 ± 1.00	< LLOQ	86 ± 20
Amitriptyline	1.85 ± 0.30	0.47 ± 0.20	1.08 ± 0.30	D	32 ± 8
Amlodipine	< LLOQ	0.22 ± 0.10	0.92 ± 0.30	14 ± 5	-
Amphetamine	0.61 ± 0.1	< LLOQ	-	D	-
Antipyrine	0.1 ± 0.1	0.05 ± 0.02	0.08 ± 0.03	9.1 ± 5.0	-
Atorvastatin	0.27 ± 0.05	D	2.98 ± 1.00	-	35 ± 20
Atrazine	0.85 ± 0.40	0.56 ± 0.30	0.78 ± 0.30	-	12 ± 2
Azithromycin	-	0.21 ± 0.05	D	D	160 ± 40
Azoxystrobin	0.1 ± 0.07	0.15 ± 0.20	0.11 ± 0.07	< LLOQ	-
Benzatropine	-	-	-	7.3 ± 1.0	-
Benzoylecgonine	0.4 ± 0.2	0.08 ± 0.04	0.34 ± 0.20	-	20 ± 7
Bezafibrate	0.82 ± 0.20	0.63 ± 0.20	0.84 ± 0.40	-	-
Bisoprolol	1 ± 0.5	0.44 ± 0.20	1.11 ± 1.00	-	19 ± 5
Bupropion	0.05 ± 0.02	< LLOQ	< LLOQ	-	-
Buspirone	< LLOQ	0.07 ± 0.01	0.1 ± 0.02	-	-
Carbamazepine	-	D	D	12.2 ± 7.0	333 ± 100
Carbamazepine-10,11-epoxide	4.23 ± 2.00	2.06 ± 0.50	4.2 ± 2.0	< LLOQ	47 ± 9
Celecoxib	< LLOQ	0.06 ± 0.03	0.24 ± 0.09	-	-
Citalopram	D	2.75 ± 2.00	D	100.2 ± 14.0	259 ± 60
Clarithromycin	D	0.96 ± 1.00	D	9.2 ± 3.0	121 ± 50
Clopidogrel	1.51 ± 0.40	1.31 ± 1.00	1.9 ± 1.4	7.2 ± 2.0	29 ± 7
Clothianidin	< LLOQ	-	-	49.5 ± 40.0	-
Clozapine	D	1.21 ± 0.40	D	11.9 ± 6.0	21 ± 5
Cocaine	0.22 ± 0.08	0.41 ± 0.60	0.29 ± 0.20	< LLOQ	22 ± 20
Cyromazine	-	-	1.62 ± 0.20	-	30 ± 6
Diazepam	0.10 ± 0.03	-	0.10 ± 0.02	-	-
Diclofenac	D	D	D	10.5 ± 5.0	266 ± 40
Diphenhydramine	2.25 ± 0.60	0.65 ± 0.40	D	11.8 ± 3.0	53 ± 100
Fluoxetine	0.78 ± 0.30	0.27 ± 0.10	D	33.3 ± 14.0	15 ± 2
Flurbiprofen	2.34 ± 0.50	-	2.41 ± 0.40	-	-
Hydrochlorothiazide	1.58 ± 0.40	0.85 ± 0.20	2.05 ± 0.80	-	-
Imidacloprid	4.54 ± 2.00	4.71 ± 0.20	3.73 ± 2.00	64 ± 13	66 ± 20
Isocarbamid	-	0.2 ± 0.09	-	D	-
Ketamine	D	1.97 ± 1.00	D	< LLOQ	46 ± 10
Ketoconazole	D	< LLOQ	D	17.8 ± 4.0	-
Levamisole	1.47 ± 0.80	0.93 ± 0.50	3.32 ± 1.00	-	53 ± 30
Levocabastine	-	-	0.09 ± 0.01	-	-
Lidocaine	D	2.09 ± 0.80	D	21.2 ± 17.0	100 ± 30

Table S2. Continued

Compounds	HLB (ng disk^{-1})	MM-anion (ng disk^{-1})	MM-cation (ng disk^{-1})	G. pulex (ng g^{-1})	H_2O (ng L^{-1})
Lincomycin	0.15 ± 0.03	0.04 ± 0.01	0.10 ± 0.07	6.8 ± 2.0	-
Lorazepam	< LLOQ	-	1.54 ± 0.40	-	-
MDMA	1.02 ± 0.60	0.21 ± 0.09	D	-	26 ± 20
Medroxyprogesterone	1.75 ± 0.80	0.75 ± 0.30	0.7 ± 0.14	-	-
Mefenamic acid	D	D	D	D	32 ± 10
Memantine	0.96 ± 0.30	0.27 ± 0.10	2.5 ± 0.90	-	19 ± 3
Mephedrone	0.02 ± 0.02	< LLOQ	0.3 ± 0.30	-	< LLOQ
Methamphetamine	0.75 ± 0.60	0.12 ± 0.05	D	< LLOQ	20 ± 6
Methylphenidate	0.1 ± 0.06	0.04 ± 0.01	0.13 ± 0.04	-	-
Metoprolol	0.2 ± 0.07	0.08 ± 0.04	0.33 ± 0.20	-	12 ± 1
Nadolol	< LLOQ	-	0.04 ± 0.01	-	-
Nicotine	2.74 ± 0.50	1.92 ± 0.70	D	D	240 ± 400
Nordiazepam	0.34 ± 0.10	0.36 ± 0.20	0.43 ± 0.20	-	-
Nortriptyline	0.42 ± 0.10	0.14 ± 0.10	0.58 ± 0.30	7.5 ± 3.0	< LLOQ
Orphenadrine	0.16 ± 0.04	0.05 ± 0.02	0.11 ± 0.05	< LLOQ	-
Oxamyl	-	-	-	17.9 ± 13.0	-
Oxazepam	0.8 ± 0.30	0.77 ± 0.40	4.48 ± 0.40	< LLOQ	< LLOQ
Oxycodone	0.28 ± 0.10	0.12 ± 0.04	0.3 ± 0.1	-	-
Pirenzipine	0.44 ± 0.20	0.09 ± 0.10	0.74 ± 0.40	-	14 ± 2
Prometryn	0.2 ± 0.1	-	< LLOQ	-	-
Propamocarb	0.06 ± 0.01	-	< LLOQ	-	< LLOQ
Propranolol	4.65 ± 0.60	2.27 ± 0.40	0.98 ± 0.70	8.2 ± 2.0	50 ± 9
Propazine	0.19 ± 0.05	< LLOQ	0.18 ± 0.02	-	-
Risperidone	< LLOQ	-	0.09 ± 0.03	6.6 ± 0.1	21 ± 8
Rizatriptan	-	-	0.03 ± 0.01	-	-
Roxithromycin	0.10 ± 0.06	0.05 ± 0.02	0.10 ± 0.05	-	-
Salbutamol	0.34 ± 0.10	0.06 ± 0.04	0.82 ± 0.50	-	12 ± 1
Salicylic acid	D	D	D	D	294 ± 200
Sertraline	2.77 ± 0.40	1.14 ± 0.50	D	D	D
Simazine	0.64 ± 0.05	0.22 ± 0.10	0.34 ± 0.10	-	-
Spiramycin	D	0.22 ± 0.20	< LLOQ	D	-
Sulfadimethoxine	0.05 ± 0.01	0.13 ± 0.04	-	< LLOQ	-
Sulfamethazine	< LLOQ	0.05 ± 0.01	0.05 ± 0.03	-	-
Sulfamethoxazole	2.88 ± 0.80	1.51 ± 0.50	1.86 ± 0.40	17 ± 14.0	34 ± 20
Sulfamonomethoxine	0.59 ± 0.10	0.49 ± 0.20	0.36 ± 0.1	-	< LLOQ
Sulfapyridine	D	1.97 ± 1.00	D	5.5 ± 2.0	104 ± 50
Sulfathiazole	-	-	-	20.8 ± 17.0	-
Tamsulosin	0.13 ± 0.09	0.12 ± 0.10	0.16 ± 0.09	-	-
Temazepam	1.46 ± 0.60	1.18 ± 0.70	5.03 ± 0.60	< LLOQ	22 ± 5
Terbutryn	0.28 ± 0.06	0.52 ± 0.50	1.48 ± 0.80	-	14 ± 4

Table S2. Continued

**Compounds**	**HLB (ng disk^{-1})**	**MM-anion (ng disk^{-1})**	**MM-cation (ng disk^{-1})**	***G. pulex* (ng g^{-1})**	**H_2O (ng L^{-1})**
Terfenadine	-	0.22 ± 0.04	-	-	-
Thiacloprid	0.03 ± 0.01	2.24 ± 2.00	0.02 ± 0.001	< LLOQ	-
Thiamethoxam	0.09 ± 0.01	< LLOQ	-	-	-
Timolol	0.13 ± 0.10	0.15 ± 0.20	0.13 ± 0.07	-	-
Tramadol	D	0.47 ± 0.05	D	< LLOQ	246 ± 50
Trimethoprim	D	3.36 ± 0.20	D	6.1 ± 2.0	95 ± 20
Valsartan	0.55 ± 0.09	1.05 ± 1.00	0.35 ± 0.10	-	-
Venlafaxine	D	2.31 ± 2.00	D	6.9 ± 2.0	178 ± 30
Verapamil	0.29 ± 0.30	0.23 ± 0.10	0.44 ± 0.40	< LLOQ	17 ± 2
Warfarin	0.14 ± 0.07	0.17 ± 0.03	0.11 ± 0.05	11.6 ± 7.0	< LLOQ

< LLOQ: concentrations below LLOQ; D: compound detected but not quantifiable; - not detected.
Refer to Table S3 for method performance data for the MM-anion, MM-cation, and *G. pulex*. For HLB method performance, refer to (Richardson et al., 2022).
Refer to Table S4 for MM-anion and MM-cation recovery data.

Table S3. Selected method performance data of the analysis of passive sampler extracts from the *G. pulex*, MM-anion, and MM-cation sorbent phases. Weekly LOD and LLOQs were calculated using the determined R_s value over an exposure period of seven days for the 3D-PSD sorbents, refer to (Richardson et al., 2022) for additional details.

Compound	MM-anion					MM-cation					*G. pulex*		
	LIN	LOD		LLOQ		LIN	LOD		LLOQ		LIN	LOD	LLOQ
	(R^2)	pg disk^{-1}	Weekly ng L^{-1}	pg disk^{-1}	Weekly ng L^{-1}	(R^2)	pg disk^{-1}	Weekly ng L^{-1}	pg disk^{-1}	Weekly ng L^{-1}	(R^2)	(ng g^{-1})	(ng g^{-1})
2-(Thiocyanomethylthio)benzothiazole	0.995	95	-	285	-	0.985	174	-	523	-	-	-	-
4-Fluoromethacationone	0.994	87	-	261	-	-	-	-	-	-	0.600	5.2	15.6
4-Methylethcathinone	0.993	18	0.8	55	2.3	0.991	55	0.2	165	0.5	0.982	1.2	3.7
Acetamiprid	0.998	11	0.2	34	0.5	0.999	6	0.1	17	0.2	0.985	1.3	3.8
Alprazolam	0.997	32	0.4	97	1.1	0.974	45	0.4	136	1.3	0.935	1.7	5.1
Ametryn	0.981	30	0.7	89	2.1	0.982	80	0.5	239	1.5	0.979	1.3	4
Amiodarone	-	-	-	-	-	0.250	259	-	777	-	-	-	-
Amitriptyline	0.993	12	0.7	36	2.2	0.995	11	0.1	32	0.2	-	-	-
Amlodipine	0.996	78	-	233	-	0.983	36	-	109	-	0.970	1.4	4.3
Amphetamine	0.993	100	12.5	299	37.4	0.988	74	-	221	-	-	-	-
Antipyrine	0.995	11	0.3	32	0.9	0.986	18	0.2	54	0.6	0.996	1.1	3.4
Atorvastatin	0.989	144	3.1	433	9.3	0.982	191	-	573	-	0.915	1.9	5.6
Atrazine	0.985	145	-	436	-	0.998	62	0.3	187	0.9	0.993	1.2	3.6
Azithromycin	0.999	33	-	100	-	0.990	27	0.2	82	0.5	0.660	4.2	12.7
Azoxystrobin	0.998	11	0.2	32	0.5	0.995	11	0.2	33	0.5	0.981	1.3	4
Benoxacor	0.803	707	-	2121	-	-	211	-	632	-	0.340	0.1	0.2
Bensulide	0.996	76	-	229	-	0.996	72	-	215	-	-	-	-
Benzatropine	0.980	22	0.6	65	1.8	0.989	16	0.1	48	0.2	0.933	1.5	4.5
Benzoylecgonine	0.991	14	1.1	42	3.2	0.996	13	0.6	40	1.9	0.994	1.2	3.5
Betaxolol	0.994	11	0.3	34	1	0.997	15	0.1	46	0.3	0.917	1.7	5.1
Bezafibrate	0.983	20	0.2	60	0.6	0.998	6	0.1	18	0.4	0.907	1.5	4.5
Bisoprolol	0.993	13	0.3	38	0.9	0.999	5	0.03	16	0.1	0.986	1.3	3.8
Bupropion	0.996	76	1.8	229	5.3	0.997	69	0.3	208	0.9	0.981	1.3	4
Buspirone	0.998	6	0.1	19	0.3	0.982	20	0.1	60	0.3	0.991	1.2	3.6
Carazolol	0.999	6	-	17	-	0.997	15	0.1	45	0.2	0.971	1.4	4.3
Carbamazepine	0.996	75	0.9	224	2.8	0.996	9	0.1	28	0.3	0.878	2.1	6.4
Carbamazepine-10,11-epoxide	0.986	26	-	78	-	0.992	103	0.6	310	1.9	0.996	1.1	3.4
Carboxine	0.983	77	-	231	-	0.997	85	-	255	-	0.957	1.3	3.8
Carfentrazone-ethyl	0.990	21	-	63	-	0.996	76	-	229	-	0.796	2.2	6.6
Celecoxib	0.995	11	-	33	-	0.985	26	0.3	78	1	0.965	1.5	4.4
Chloramphenicol	0.981	83	-	248	-	0.979	22	-	66	-	-	-	-
Cilazapril	0.999	6	0.1	19	0.4	0.997	16	0.7	48	2.1	0.989	1.2	3.7
Citalopram	0.999	7	-	20	-	0.995	11	-	32	-	0.736	3.3	10
Clarithromycin	0.994	16	-	49	-	0.979	22	0.1	65	0.2	0.952	1.6	4.7
Clodinafop-propargyl	0.996	38	-	113	-	0.977	219	-	657	-	0.890	2	6.1
Clofibric acid	0.983	154	-	461	-	0.887	54	-	161	-	0.837	2.4	7.3
Clopidogrel	0.992	20	0.4	59	1.2	0.989	63	0.8	189	2.5	0.984	1.3	3.9
Clothianidin	0.993	13	0.2	39	0.6	0.999	10	0.1	30	0.3	0.688	2.5	7.6
Clozapine	0.995	88	3.8	264	11.5	-	-	-	-	-	0.880	2.1	6.3
Cocaine	0.999	9	0.1	27	0.3	0.994	11	0.1	34	0.2	0.978	1.4	4.1
Cyclouron	0.989	132	-	397	-	0.992	146	-	437	-	0.937	1.7	5.1
Cycloxyidim	-	-	-	-	-	-	492	-	1477	-	-	-	-
Cymoxanil	0.991	132	-	395	-	-	-	-	-	-	-	-	-
Cyromazine	0.992	119	-	357	-	0.976	210	-	630	-	-	-	-
Diazepam	0.991	111	1.3	332	3.8	0.981	163	1.8	489	5.5	0.988	1.2	3.7
Diclofenac	0.991	111	1.5	333	4.4	0.985	18	0.5	55	1.5	0.910	1.9	5.7
Diflubenzuron	0.994	12	-	36	-	0.987	17	-	52	-	0.860	2.3	6.8
Dimethametryn	0.984	19	-	58	-	0.977	42	-	127	-	0.880	2.1	6.3
Dimethomorph	0.992	108	-	323	-	0.988	24	-	72	-	0.963	1.5	4.5
Diphenhydramine	0.997	8	1.3	25	4	0.992	14	0.1	41	0.2	0.930	1.7	5.2

Table S3. Continued

Compound	MM-anion					MM-cation					*G. pulex*		
	LIN	LOD		LLOQ		LIN	LOD		LLOQ		LIN	LOD	LLOQ
	(R^2)	pg disk^{-1}	Weekly ng L^{-1}	pg disk^{-1}	Weekly ng L^{-1}	(R^2)	pg disk^{-1}	Weekly ng L^{-1}	pg disk^{-1}	Weekly ng L^{-1}	(R^2)	(ng g^{-1})	(ng g^{-1})
Enalapril	0.997	59	-	176	-	0.984	27	-	81	-	0.881	2.1	6.3
Famoxadone	0.984	19	-	57	-	0.995	88	-	264	-	0.849	2.3	7
Fenoxaprop-ethyl	0.012	12757	-	38270	-	-	-	-	-	-	0.370	6.1	18.3
Fenuron	0.980	31	-	92	-	0.993	95	-	284	-	0.994	1.1	3.4
Fluocinonide	-	82	-	245	-	0.967	262	-	786	-	0.948	1.3	4
Fluoxetine	0.993	17	2.1	52	6.2	0.986	18	-	54	-	0.976	1.4	4.1
Flurbiprofen	-	-	-	-	-	-	-	-	-	-	-	-	-
Flurochloridone	0.997	70	-	211	-	0.980	167	-	500	-	0.836	2.2	6.6
Flutamide	0.998	10	0.1	29	0.4	0.998	12	0.2	35	0.5	0.982	1.3	3.8
Flutolanil	0.997	8	-	23	-	0.995	16	-	47	-	0.860	2.1	6.2
Fuberidazole	0.999	6	-	18	-	0.998	10	-	31	-	0.871	2.2	6.5
Gemfibrozil	-	-	-	-	-	0.869	450	-	1350	-	-	-	-
Hydrochlorothiazide	0.985	72	-	217	-	0.989	29	-	86	-	0.813	2.6	7.9
Imidacloprid	0.986	33	0.3	98	1	0.984	19	0.2	58	0.7	0.847	2.4	7.1
Isocarbamid	0.988	25	-	74	-	0.996	70	-	210	-	0.604	4.2	12.7
Isradipine	0.901	76	-	227	-	0.852	116	-	348	-	0.942	1.4	4.2
Josamycin	0.997	12	-	36	-	0.989	155	-	466	-	0.983	1.3	3.9
Ketamine	0.983	28	0.4	84	1.1	0.987	67	0.2	200	0.5	0.992	1.2	3.6
Ketoconazole	0.991	112	-	337	-	-	-	-	-	-	0.695	3.8	11.4
Ketoprofen	0.940	335	-	1005	-	0.007	-	-	-	-	-	-	-
Ketotifen	0.994	11	0.4	33	1.2	0.997	9	0.1	27	0.2	0.887	2.1	6.2
Levamisole	0.995	14	0.5	43	1.5	0.996	10	0.1	29	0.2	-	-	-
Levocabastine	0.995	16	0.1	49	0.4	0.993	23	0.2	68	0.6	0.981	1.3	4
Lidocaine	0.997	12	0.2	35	0.7	0.991	14	0.1	42	0.2	0.997	1.1	3.3
Lincomycin	0.999	24	-	72	-	0.999	7	0.2	22	0.7	0.996	1.1	3.4
Lorazepam	0.994	95	-	285	-	-	205	-	616	-	0.986	1.3	3.8
MDMA	0.999	8	1.2	25	3.6	0.985	27	-	80	-	0.993	1.2	3.5
Meclizine	0.987	137	-	411	-	0.068	5301	-	15904	-	0.507	7.3	21.9
Medroxyprogesterone	0.991	26	-	79	-	0.997	11	-	33	-	0.976	1.4	4.1
Mefenamic acid	0.990	128	-	383	-	0.957	59	1.9	178	5.7	-	-	-
Memantine	0.997	13	0.8	38	2.5	0.989	62	0.1	187	0.4	0.961	1.5	4.5
Mephedrone	0.995	15	3.6	46	10.7	0.993	48	0.2	145	0.6	0.997	1.1	3.4
Mephosfolan	0.999	11	-	32	-	0.997	8	-	23	-	0.859	2.3	6.8
Metformin	0.987	135	-	405	-	0.701	858	-	2574	-	-	-	-
Methamphetamine	0.993	18	4.9	55	14.8	0.986	71	-	213	-	0.984	1.3	3.9
Methcathinone	0.993	18	6.8	55	20.4	0.995	16	0.03	48	0.1	0.973	1.4	4.2
Methedrone	0.990	15	3.1	44	9.2	0.978	42	0.2	127	0.7	0.909	1.6	4.9
Methylphenidate	0.999	10	-	31	-	0.982	20	0.1	60	0.2	0.993	1.2	3.6
Metoprolol	0.998	12	0.4	36	1.2	0.997	12	0.1	35	0.2	0.981	1.3	4
Morphine	0.985	34	-	102	-	0.977	90	-	269	-	0.951	1.4	4.3
Nadolol	0.997	12	0.7	37	2.1	0.999	9	0.1	26	0.2	0.991	1.2	3.7
Nicotine	0.991	115	-	346	-	-	-	-	-	-	-	-	-
Nifedipine	0.979	95	-	285	-	0.991	114	-	341	-	0.851	1.6	4.8
Nitenpyram	0.999	10	1	29	3	0.991	75	-	225	-	0.967	1.5	4.4
Nordiazepam	0.995	20	0.4	59	1.3	0.990	15	0.3	44	0.9	0.983	1.3	3.9
Norethisterone	0.999	45	-	136	-	-	54	-	162	-	-	-	-
Nortriptyline	0.994	17	2.3	50	7	0.977	23	0.1	69	0.3	0.983	1.2	3.5
Orphenadrine	0.999	5	0.5	16	1.6	0.995	11	0.1	33	0.2	0.825	1.8	5.4
Oxamyl	0.998	25	8.2	76	24.6	0.997	15	-	44	-	0.992	1.2	3.6
Oxazepam	0.996	14	0.2	43	0.7	0.995	20	0.8	59	2.5	0.986	1.3	3.8
Oxycarboxin	0.999	10	-	31	-	0.986	32	-	96	-	0.836	2.4	7.3

Table S3. Continued

Compound	MM-anion					MM-cation					*G. pulex*		
	LIN	LOD		LLOQ		LIN	LOD		LLOQ		LIN	LOD	LLOQ
	(R^2)	pg disk^{-1}	Weekly ng L^{-1}	pg disk^{-1}	Weekly ng L^{-1}	(R^2)	pg disk^{-1}	Weekly ng L^{-1}	pg disk^{-1}	Weekly ng L^{-1}	(R^2)	(ng g^{-1})	(ng g^{-1})
Oxycodone	0.999	5	0.2	14	0.6	0.999	5	0	14	0.1	0.937	1.7	5.1
Picoxystrobin	0.998	59	1.1	177	3.4	0.989	30	-	90	-	0.969	1.4	4.3
Piperophos	0.996	9	-	27	-	0.993	13	-	39	-	0.893	2	6
Pirenzipine	0.999	4	0.4	11	1.3	0.996	18	0.3	54	1	0.995	1.2	3.5
Pretilachlor	0.993	100	-	301	-	0.989	124	-	372	-	0.764	2.2	6.5
Prodiamine	-	-	-	-	-	0.886	487	-	1462	-	-	-	-
Prometon	0.982	29	0.5	86	1.5	0.997	37	0.2	110	0.7	0.996	1.1	3.4
Prometryn	0.996	77	1.3	232	4	0.987	74	0.4	222	1.1	0.988	1.2	3.7
Propamocarb	0.998	9	-	28	-	0.986	18	0.1	53	0.3	0.912	1.9	5.6
Propranolol	0.999	5	0.2	16	0.5	0.976	24	0.1	71	0.3	0.981	1.3	4
Propazine	0.984	153	-	459	-	0.998	57	0.3	171	0.8	0.989	1.2	3.6
Pymetrozine	0.999	5	0.8	14	2.3	0.999	6	0.2	17	0.7	0.951	1.6	4.7
Pyracarbolid	0.991	21	-	62	-	0.984	35	-	106	-	0.980	1.3	3.9
Pyraclostrobin	0.993	110	-	331	-	0.999	36	-	109	-	0.934	1.4	4.3
Pyraflufen-ethyl	0.984	36	-	108	-	0.997	73	-	219	-	0.585	3.2	9.6
Pyridaben	-	-	-	-	-	0.986	79	-	237	-	-	-	-
Risperidone	0.994	99	2.9	296	8.6	-	-	-	-	-	0.967	1.3	4
Rizatriptan	0.993	13	-	39	-	0.986	71	1.1	214	3.2	0.984	1.3	3.9
Ronidazole	0.999	7	1.2	22	3.5	0.999	6	0.2	19	0.7	0.975	1.4	4.1
Roxithromycin	0.998	7	-	21	-	0.995	11	-	32	-	0.967	1.3	4
Salbutamol	0.999	7	3.2	20	9.5	0.999	10	0.1	30	0.3	0.971	1.4	4.2
Salicylic acid	0.552	1046	-	3138	-	0.469	159	-	478	-	-	-	-
Sertraline	0.981	30	-	90	-	0.878	56	-	167	-	-	-	-
Simazine	0.987	155	-	465	-	0.990	119	0.6	357	1.9	0.985	1.3	3.9
Spinosyn A	0.995	83	-	250	-	0.917	45	-	135	-	0.961	1.4	4.3
Spinosyn D	0.809	73	-	218	-	0.984	19	-	57	-	0.936	1.4	4.3
Spiramycin	0.981	21	-	62	-		-	-	-	-	0.773	3	9
Sulfadimethoxine	0.996	9	0.1	27	0.2	0.997	16	0.6	48	1.7	0.982	1.3	3.9
Sulfamerazine	0.997	85	-	255	-		-	-	-	-	-	-	-
Sulfamethazine	0.993	13	0.3	38	0.8	0.999	5	0.4	15	1.3	0.989	1.2	3.7
Sulfamethoxazole	0.993	60	0.8	180	2.5	0.989	30	2.2	90	6.7	0.994	1.1	3.3
Sulfamonomethoxine	0.985	73	-	218	-	0.995	81	4.6	244	13.9	0.992	1.2	3.6
Sulfapyridine	0.995	11	3.6	32	10.8	0.996	9	0.4	27	1.2	0.987	1.3	3.8
Sulfathiazole	0.988	31	3.3	92	9.9	0.999	3	0.2	10	0.6	0.966	1.5	4.4
Sulfisoxazole	0.989	23	0.4	68	1.3	0.996	10	-	30	-	0.990	1.2	3.7
Tacrine	0.996	10	0.4	30	1.3	0.978	41	-	122	-	0.988	1.3	3.8
Tamsulosin	0.998	11	0.2	34	0.7	0.990	15	0.1	44	0.3	0.988	1.2	3.7
Temazepam	0.995	11	0.2	33	0.6	-	6	0.1	19	0.4	0.99	1.2	3.7
Terbutryn	0.996	75	1	226	3.1	0.987	73	0.3	220	1	-	-	-
Terfenadine	0.959	31	-	92	-	0.991	14	-	43	-	0.950	1.6	4.8
Thiacloprid	0.997	15	0.1	44	0.3	0.997	8	0.1	23	0.2	0.974	1.4	4.2
Thiamethoxam	0.997	15	0.3	45	0.8	0.976	35	0.7	105	2.2	0.947	1.6	4.8
Thiazopyr	0.986	143	-	430	-	0.985	143	-	429	-	0.851	2.3	7
Timolol	0.999	8	0.2	23	0.6	0.999	5	0.03	16	0.1	0.991	1.2	3.6
Tramadol	0.998	11	0.3	32	0.9	0.996	10	0.04	29	0.1	0.922	1.8	5.4
Trimethoprim	0.996	18	0.6	53	1.9	0.998	6	0.1	19	0.2	0.978	1.4	4.1
Valsartan	0.988	141	3	423	8.9	0.994	92	7.3	275	21.8	0.976	1.2	3.7
Venlafaxine	0.990	15	0.8	45	2.3	0.999	5	0	16	0.1	0.973	1.4	4.2
Verapamil	0.998	7	0.1	22	0.3	0.993	13	0.1	38	0.2	0.983	1.3	3.9
Warfarin	0.997	66	1.2	199	3.6	0.998	13	0.4	38	1.2	0.986	1.3	3.8
Ziprasidone	0.996	74	-	221	-	0.976	35	-	106	-	0.935	1.7	5.1

Table S4. Comparison of the mean (standard deviation, *n* = 9) percentage recovery of each compound using the MM-anion and MM-cation sorbents. Refer to Richardson *et al.*, 2022 for HLB recovery data.

Compound	MM-anion	MM-cation	Compound	MM-anion	MM-cation	Compound	MM-anion	MM-cation
2-(Thiocyanomethylthio)benzothiazole	48 (21)	2 (1)	Carazolol	62 (13)	40 (7)	Fenoxaprop-ethyl	27 (16)	2 (0)
4-Fluoromethacationone	43 (13)	24 (8)	Carbamazepine	75 (9)	76 (10)	Fenuron	50 (14)	50 (17)
4-Methylethcathinone	60 (11)	25 (14)	Carboxine	3 (4)	12 (8)	Flufenoxuron	10 (5)	44 (22)
Acetamiprid	72 (9)	70 (8)	Carfentrazone-ethyl	61 (11)	2 (1)	Fluocinonide	83 (11)	40 (17)
Aclonifen	93 (8)	98 (9)	CBZ epoxide	49 (29)	15 (1)	Fluoxetine	83 (29)	29 (9)
Alprazolam	54 (14)	55 (13)	Celecoxib	55 (10)	61 (10)	Flurochloridone	61 (11)	45 (13)
Ametryn	50 (10)	42 (13)	Chloramphenicol	65 (16)	76 (15)	Flutamide	70 (9)	80 (11)
Amiodarone	44 (7)	27 (14)	Cilazapril	70 (8)	76 (8)	Flutolanil	68 (8)	73 (7)
Amitriptyline	87 (26)	32 (10)	Citalopram	79 (13)	52 (10)	Fuberidazole	67 (7)	62 (6)
Amlodipine	39 (11)	16 (5)	Clarithromycin	59 (33)	15 (7)	Hydrochlorothiazide	39 (5)	72 (10)
Amphetamine	57 (8)	28 (15)	Clodinafop-propargyl	60 (9)	16 (2)	Imidacloprid	74 (9)	77 (13)
Antipyrine	60 (8)	68 (12)	Clopidogrel	64 (8)	56 (10)	Isocarbamid	63 (11)	53 (9)
Atorvastatin	12 (6)	15 (6)	Clothianidin	63 (11)	61 (15)	Isradipine	39 (11)	52 (13)
Atrazine	35 (10)	44 (18)	Clothianidin-d3	45 (33)	80 (51)	Josamycin	23 (10)	9 (3)
Azelnidipine	3 (2)	31 (24)	Clozapine	45 (18)	43 (35)	Ketamine	56 (9)	38 (19)
Azithromycin	48 (27)	15 (8)	Cocaine	74 (9)	54 (10)	Ketoconazole	38 (17)	39 (25)
Azoxystrobin	70 (9)	73 (7)	Cyclouron	45 (14)	42 (21)	Ketotifen	73 (11)	45 (11)
Benoxacor	33 (21)	29 (24)	Cymoxanil	162 (8)	36 (12)	Levamisole	75 (13)	49 (14)
Bensulide	39 (20)	53 (14)	Diazepam	65 (10)	73 (8)	Levocabastine	75 (10)	71 (8)
Benzatropine	85 (27)	45 (11)	Diclofenac	55 (13)	72 (8)	Lidocaine	59 (8)	42 (18)
Benzoylecgonine	66 (8)	72 (13)	Diflubenzuron	63 (12)	68 (8)	Lincomycin	25 (15)	42 (9)
Betaxolol	66 (9)	56 (9)	Dimethametryn	61 (8)	47 (9)	Lorazepam	78 (10)	71 (14)
Bezafibrate	73 (10)	83 (11)	Dimethomorph	77 (8)	73 (11)	MDMA	60 (6)	45 (51)
Bisoprolol	71 (9)	59 (11)	Diphenhydramine	78 (12)	33 (11)	Meclizine	37 (21)	42 (24)
Bupropion	47 (16)	19 (14)	Enalapril	73 (11)	77 (10)	Medroxyprogesterone	77 (9)	77 (9)
Buspirone	49 (7)	44 (12)	Famoxadone	51 (17)	59 (6)	Mefenamic acid	32 (9)	59 (10)

Table S4. Continued

Compound	MM-anion	MM-cation	Compound	MM-anion	MM-cation	Compound	MM-anion	MM-cation
Memantine	63 (8)	28 (12)	Pirenzipine	27 (4)	44 (18)	Sulfadimethoxine	17 (9)	72 (8)
Mephedrone	50 (12)	18 (12)	Pretilachlor	44 (6)	44 (16)	Sulfamerazine	85 (20)	44 (12)
Mephosfolan	68 (12)	72 (7)	Prodiamine	20 (11)	25 (13)	Sulfamethazine	14 (9)	69 (8)
Metformin	25 (16)	8 (2)	Prometon	51 (13)	45 (19)	Sulfamethoxazole	15 (9)	69 (9)
Methamphetamine	55 (7)	26 (12)	Prometryn	49 (10)	38 (14)	Sulfamonomethoxine	48 (12)	89 (18)
Methcathinone	53 (18)	18 (9)	Propamocarb	106 (16)	44 (14)	Sulfapyridine	9 (6)	58 (15)
Methedrone	51 (8)	29 (14)	Propanolol	67 (8)	47 (8)	Sulfathiazole	8 (4)	52 (11)
Methylphenidate	69 (7)	35 (13)	Propazine	35 (10)	39 (18)	Sulfisoxazole	16 (9)	62 (10)
Metoprolol	69 (8)	55 (14)	Pymetrozine	73 (14)	75 (6)	Tacrine	73 (8)	30 (11)
Morphine	109 (27)	62 (15)	Pyracarbolid	28 (17)	11 (2)	Tamsulosin	67 (8)	63 (8)
Nadolol	51 (8)	58 (6)	Pyraclostrobin	51 (10)	68 (12)	Temazepam	66 (10)	71 (8)
Nicotine	33 (19)	30 (30)	Pyraflufen-ethyl	58 (11)	3 (1)	Terbutryn	51 (10)	37 (12)
Nifedipine	25 (14)	32 (12)	Pyridaben	28 (3)	48 (11)	Terfenadine	72 (29)	37 (9)
Nitenpyram	29 (9)	58 (7)	Risperidone	42 (15)	36 (27)	Thiacloprid	70 (8)	71 (8)
Nordiazepam	69 (10)	71 (13)	Rizatriptan	20 (3)	34 (13)	Thiamethoxam	54 (6)	57 (6)
Norethisterone	71 (9)	79 (20)	Ronidazole	64 (8)	73 (8)	Thiazopyr	33 (14)	32 (16)
Nortriptyline	72 (20)	23 (10)	Roxithromycin	52 (31)	14 (5)	Timolol	49 (10)	50 (10)
Orphenadrine	84 (16)	33 (11)	Salbutamol	102 (40)	67 (8)	Tramadol	74 (8)	44 (16)
Oxamyl	54 (8)	19 (11)	Salicylic acid	57 (16)	103 (18)	Trimethoprim	59 (9)	55 (6)
Oxazepam	62 (8)	65 (7)	Sertraline	85 (27)	40 (11)	Valsartan	37 (13)	73 (10)
Oxycarboxin	66 (9)	2 (2)	Simazine	36 (7)	46 (16)	Venlafaxine	72 (9)	49 (14)
Oxycodone	44 (7)	41 (15)	Spinosyn A	58 (39)	24 (12)	Verapamil	66 (9)	44 (12)
Picoxystrobin	55 (8)	56 (10)	Spinosyn D	58 (47)	27 (15)	Warfarin	65 (6)	73 (9)
Piperophos	46 (16)	60 (7)	Spiramycin	12 (8)	1 (1)	Ziprasidone	59 (17)	20 (16)

S5. Calibration of the 3D-PSD for the anion and cation phases

The sampling rates (R_s) for the 9 mm MM-anion and MM-cation phases were determined as described previously (Richardson et al., 2022). In summary, 5 L of artificial freshwater (AFW, (OECD, 2019)) was fortified to 100 ng L^{-1} with a mix of 164 analytical standards and agitated using a magnetic stirrer. Twelve fully assembled 3D-PSDs per sorbent phase were exposed within the beaker and a single device was removed at the following timepoints: 8, 24, 48, 72, 96, 120, 144, and 168 hours. A sample of water was taken from the beaker coinciding with the 3D-PSD collection and the fortified AFW was replaced after sampling. An unused, fully assembled 3D-PSD was exposed to the air and underwent the same handling to act as a negative control. The 9 mm sorbent disks were prepared for analysis as described in the methods. Water samples were prepared for LC-MS analysis as described previously (Ng et al., 2020; Richardson et al., 2022). Briefly, 900 µL of river water sample was spiked with 100 µL of SIL-IS in MeOH (500 ng L^{-1}). After vortexing, water samples were filtered (0.2 µm PTFE) directly into deactivated HPLC vials. Sample quantification was done with a 14-point external AFW calibration series. Calibrants were prepared as above with the 900 µL of AFW water spiked with 100 µL of MeOH containing analytical standards to a final concentration over the range of 5 to 2,000 ng L^{-1} with the SIL-IS concentration remaining to 500 ng L^{-1} for all samples.

The R_s values were determined as the ratio between the slope of the regression line representing the contaminant mass on the sorbent disk per unit of time and the mean concentration in the fortified AFW over the same time period (Eqn. S1) (Castle et al., 2018; Vrana et al., 2005). Refer to Table S1 for R_s values determined on the MM-anion and MM-cation phases.

$$R_s = \frac{C_s}{C_w.t} \qquad \text{Eqn. S1}$$

Table S5. Experimentally determined R_s values (± standard deviation (SD)) for 93 compounds on the 9 mm HLB, anion, and cation 9 mm phases,

Compound	R_s ± SD (mL/d)			LogP	LogD (pH 7.4)
	HLB*	MM-anion	MM-cation		
4-Methylethcathinone	-	3.4 ± 0.5	47.9 ± 1.9	2.18	1.87
Acetamiprid	-	9.4 ± 0.5	10 ± 0.8	1.06	1.06
Alprazolam	-	12.7 ± 2.6	16 ± 0.8	2.63	2.63
Ametryn	-	6 ± 2.1	27.5 ± 1.4	3.04	3.04
Amitriptyline	-	3.2 ± 0.3	22.6 ± 4.7	4.72	2.95
Amphetamine	1.6 ± 0.1	-	-	1.82	-0.62
Antipyrine	-	4.8 ± 0.3	13.7 ± 0.8	0.72	0.72
Atorvastatin	-	4.6 ± 0.3	-	4.36	1.26
Atrazine	-	-	31.8 ± 9.6	2.66	2.66
Azithromycin	-	-	25.8 ± 3.3	3.29	1.36
Azoxystrobin	10.2 ± 0.8	7.8 ± 2.8	9.5 ± 1.9	3.54	3.54
Benzatropine	3.2 ± 0.1	6.1 ± 0.1	33.8 ± 9	4.71	1.89
Benzoylecgonine	8.5 ± 0.7	1.9 ± 0.1	3.1 ± 0.3	2.29	-0.21
Betaxolol	-	5 ± 1.2	23.4 ± 3.4	2.87	0.77
Bezafibrate	-	13.7 ± 3.4	6.4 ± 1.2	3.48	-0.11
Bisoprolol	3.7 ± 0.3	5.8 ± 0.8	27.4 ± 2.7	2.21	0.12
Bupropion	-	6.2 ± 1.1	28.1 ± 2.1	3.08	2.88
Buspirone	2.4 ± 0.4	10.1 ± 3.9	33.6 ± 3.7	2.95	2.59
Carazolol	4.9 ± 0.5	-	30.3 ± 6.3	3.33	1.15
Carbamazepine	12.3 ± 1	11.1 ± 0.9	13 ± 1.4	2.28	2.28
Carbamazepine-10,11-epoxide	4.2 ± 0.4	-	23.3 ± 2	6.09	6.09
Celecoxib	-	-	10.7 ± 3	3.24	3.24
Cilazapril	-	6.2 ± 1.9	3.2 ± 0.5	1	-2.5
Citalopram	1.6 ± 0.2	-	-	3.39	1.27
Clarithromycin	-	-	60.8 ± 10.6	3.12	2.37
Clopidogrel	-	7.3 ± 2.7	11.4 ± 1.7	4.21	4.21
Clothianidin	-	9.7 ± 1	13 ± 1.4	-0.03	-0.03
Cocaine	-	12.5 ± 2.2	22 ± 3.9	2.78	1.21
Diazepam	-	12.5 ± 2.5	12.8 ± 1.7	2.92	2.92
Diclofenac	-	10.9 ± 4.3	5.4 ± 1.5	4.48	1.37
Dimethametryn	3.4 ± 0.9	-	-	3.73	3.73
Diphenhydramine	-	0.9 ± 0.3	25.4 ± 3.2	3.71	2.34
Flutamide	-	11.2 ± 2.9	10.9 ± 2.5	3.14	3.14
Flutolanil	6 ± 0.6	-	-	4.06	4.06
Fuberidazole	8.3 ± 0.6	-	-	2.54	2.54
Imidacloprid	-	14.2 ± 1.1	11.1 ± 1.2	0.81	-0.52
Isocarbamid	6.3 ± 0.5	-	-	0.65	0.65
Ketamine	2.7 ± 0	11.5 ± 0.8	53.6 ± 8.3	2.12	2.07
Ketotifen	1.3 ± 0.2	4 ± 1.3	15.7 ± 2.7	4.06	2.61

Table S5. Continued

Compound	R_s ± SD (mL/d)			LogP	LogD (pH 7.4)
	HLB*	MM-anion	MM-cation		
Levamisole	-	4.4 ± 0.2	21.6 ± 2.3	2.15	0.25
Levocabastine	-	17.4 ± 7	15.1 ± 4.4	4.48	1.98
Lidocaine	0.8 ± 0.1	7.6 ± 0.9	31.8 ± 2.4	2.33	1.81
Lincomycin	-	-	4.1 ± 0.2	0.63	-0.44
MDMA	3.3 ± 0.4	1 ± 0.1	-	1.93	-0.77
Mefenamic acid	-	-	3.9 ± 1.1	5	2.04
Memantine	5.9 ± 0.3	-	67.4 ± 3.7	3.48	0.56
Mephedrone	-	0.6 ± 0.1	37.9 ± 2	1.9	1.59
Mephosfolan	6.7 ± 0.5	-	-	1.57	1.57
Methamphetamine	1.4 ± 0.1	0.5 ± 0	-	2.17	-0.57
Methcathinone	-	0.4 ± 0	74.6 ± 6.1	1.51	1.32
Methedrone	-	-	27.9 ± 2.9	1.33	0.99
Methylphenidate	-	-	43.1 ± 3.2	2.33	0.26
Metoprolol	4.1 ± 0.4	4.2 ± 0.5	27.3 ± 1.8	1.85	-0.25
Nadolol	-	2.4 ± 0.2	18.5 ± 2.2	1.24	-0.86
Nitenpyram	-	1.4 ± 0.3	-	0.4	0.4
Nordiazepam	-	6.6 ± 1.4	6.7 ± 0.7	2.94	2.94
Nortriptyline	-	-	37.9 ± 4	4.76	2.28
Orphenadrine	-	1.4 ± 0.4	24.6 ± 3.9	4.05	2.71
Oxazepam	-	9.2 ± 1.5	3.6 ± 0.7	2.06	2.06
Oxycodone	2.8 ± 0.3	3.4 ± 0.4	16.3 ± 2.1	0.91	0.45
Picoxystrobin	-	7.3 ± 2.7	-	3.84	3.84
Pirenzipine	1 ± 0.1	1.2 ± 0.2	8.1 ± 0.8	0.31	0.03
Prometon	-	8.1 ± 2.2	26.2 ± 3.7	2.79	2.79
Prometryn	-	9.8 ± 1.1	25 ± 0.5	3.4	3.4
Propamocarb	0.3 ± 0.2	-	26.5 ± 1.6	1.15	-0.67
Propranolol	-	4.1 ± 1.4	37.4 ± 6.5	3.26	1.15
Propazine	-	-	26.7 ± 7.2	2.98	2.98
Pymetrozine	3.5 ± 0.4	-	4.3 ± 0.1	0.3	0.3
Pyracarbolid	5.4 ± 0.4	-	-	2.03	2.03
Rizatriptan	-	-	9.6 ± 0.9	1.97	0.04
Ronidazole	2.1 ± 0.2	0.9 ± 0	4 ± 0.3	-0.42	-0.42
Salbutamol	4.8 ± 0.6	0.3 ± 0	15.2 ± 1.8	0.61	-1.52
Simazine	-	-	26.7 ± 4.5	2.29	2.29
Sulfadimethoxine	-	15.3 ± 3.1	4.2 ± 0.3	1.46	-0.49
Sulfamethazine	6 ± 0.6	7 ± 0.5	1.6 ± 0.1	0.44	0.3
Sulfamethoxazole	-	10 ± 1.2	1.9 ± 0	0.65	-0.55
Sulfamonomethoxine	-	-	2.5 ± 0.2	0.56	-1.31
Sulfapyridine	6 ± 0.7	0.4 ± 0.1	3.1 ± 0.3	0.47	0.41
Sulfathiazole	-	1.3 ± 0.3	2.6 ± 0.1	0.42	0.04
Sulfisoxazole	-	7.4 ± 1.1	-	0.85	-0.77

Table S5. Continued

Compound	R_s ± SD (mL/d)			LogP	LogD (pH 7.4)
	HLB*	MM-anion	MM-cation		
Tacrine	11.9 ± 1.1	3.9 ± 0.2	-	2.87	0.81
Tamsulosin	8.1 ± 0.5	6.6 ± 2.3	20.4 ± 2.9	2.14	0.77
Temazepam	8.2 ± 0.6	8 ± 2	6.4 ± 0.9	2.11	2.11
Terbutryn	6.1 ± 1	10.4 ± 4	27.6 ± 1.3	3.35	3.35
Thiacloprid	-	18.8 ± 2.1	17.6 ± 2.2	1.22	1.22
Thiamethoxam	-	8.4 ± 0.5	6.8 ± 0.6	-0.33	-0.33
Timolol	-	5.5 ± 0.6	21.5 ± 2.1	1.53	-0.35
Tramadol	1.9 ± 0.3	5.4 ± 0.2	30.7 ± 3	2.54	0.52
Trimethoprim	6.3 ± 0.5	3.9 ± 0.8	17.3 ± 1.9	1.12	1
Valsartan	-	6.7 ± 2.5	2.2 ± 0.2	3.87	-0.86
Venlafaxine	1.6 ± 0.3	2.8 ± 0.4	23.5 ± 2.2	3.15	1.43
Verapamil	3.7 ± 0.5	10 ± 2.9	22.5 ± 5.1	3.95	2.38
Warfarin	-	11.7 ± 3.2	4.5 ± 0.7	3.1	0.31

* Adapted from Richardson *et al.* (2022) with permission from Elsevier.

Table S6. Mean concentration of contaminants detected across the 6-month study on the three 3D-PSD phases represented as time-weighted average water concentration calculated using the indicated R_s value and in water samples. Data is reported ± standard deviation and all water concentrations are in ng L^{-1} and R_s is in mL day^{-1}.

Compound	[CEC] water	HLB		MM-anion		MM-cation	
		R_s	TWA [CEC]	R_s	TWA [CEC]	R_s	TWA [CEC]
4-Methylethcathinone	-	-	-	-	-	47.9	0.4 ± 0.1
Acetamiprid	86 ± 22	-	-	9.4	34 ± 28	10	48 ± 18
Amitriptyline	32 ± 8	-	-	2.3	29 ± 13	22.6	7 ± 2
Amphetamine	-	1.6	54 ± 9	-	-	-	-
Antipyrine	-	-	-	4.8	2 ± 1	13.7	1 ± 0.3
Atrazine	12 ± 2	-	-	-	-	31.8	4 ± 1
Azithromycin	160 ± 37	-	-	-	-	-	-
Azoxystrobin	-	10.2	1 ± 1	9.3	2 ± 2	9.5	2 ± 1
Benzoylecgonine	20 ± 7	8.5	7 ± 3	1.9	6 ± 3	3.1	16 ± 7
Bezafibrate	-	-	-	13.8	7 ± 2	6.4	19 ± 9
Bisoprolol	19 ± 5	3.7	39 ± 20	5.9	11 ± 6	27.4	6 ± 6
Buspirone	-	-	-	10.1	1 ± 0.1	33.6	0.4 ± 0.1
Carbamazepine-10,11-epoxide	47 ± 9	4.2	144 ± 62	-	-	23.3	26 ± 11
Celecoxib	-	-	-	-	-	10.7	3 ± 1
Clopidogrel	29 ± 7	-	-	7.3	26 ± 19	11.4	24 ± 18
Cocaine	22 ± 19	-	-	12.7	5 ± 6	22	2 ± 1
Diazepam	-	-	-	-	-	12.8	1 ± 0.3
Diphenhydramine	53 ± 12	-	-	0.9	107 ± 57	-	-
Imidacloprid	66 ± 21	7.8	83 ± 33	14.3	47 ± 2	11.1	48 ± 20
Ketamine	46 ± 10	-	-	11	26 ± 18	-	-
Levamisole	53 ± 27	-	-	4.2	32 ± 17	21.6	22 ± 7
Levocabastine	-	-	-	-	-	15.1	1 ± 0.1
Lidocaine	100 ± 30	-	-	7.7	39 ± 14	-	-
Lincomycin	-	-	-	-	-	4.1	3 ± 2
MDMA	26 ± 17	3.3	44 ± 28	1	30 ± 13	-	-
Memantine	19 ± 3	5.9	23 ± 8	-	-	67.4	5 ± 2
Mephedrone	-	-	-	-	-	37.9	1 ± 1
Methamphetamine	20 ± 6	1.4	77 ± 57	0.5	33 ± 12	-	-
Methylphenidate	-	-	-	-	-	43.1	0.4 ± 0.1
Metoprolol	12 ± 0.4	4.1	7 ± 2	4.2	3 ± 1	27.3	2 ± 1
Nadolol	-	-	-	-	-	18.5	0.3 ± 0.1
Nordiazepam	-	-	-	-	-	6.7	9 ± 4
Nortriptyline	-	-	-	-	-	37.9	2 ± 1
Orphenadrine	-	-	-	1.4	5 ± 2	24.6	1 ± 0.3
Oxazepam	-	-	-	9.2	12 ± 6	3.6	178 ± 14
Oxycodone	-	2.8	15 ± 5	3.4	5 ± 2	16.3	3 ± 1
Pirenzipine	14 ± 2	1	63 ± 24	1.2	11 ± 12	8.1	13 ± 6
Propamocarb	-	0.3	26 ± 1	-	-	-	-

Table S6. Continued

Compound	[CEC] water	HLB		MM-anion		MM-cation	
		R_s	TWA [CEC]	R_s	TWA [CEC]	R_s	TWA [CEC]
Propranolol	50 ± 9	-	-	4.2	78 ± 15	37.4	4 ± 2
Propazine	-	-	-	-	-	26.7	1 ± 0.1
Rizatriptan	-	-	-	-	-	9.6	0.5 ± 0.1
Salbutamol	12 ± 1	4.8	10 ± 3	0.3	30 ± 20	15.2	8 ± 5
Simazine	-	-	-	-	-	26.7	2 ± 1
Sulfadimethoxine	-	-	-	15.4	1 ± 0.4	-	-
Sulfamethazine	-	-	-	7	1 ± 0.3	1.6	5 ± 2
Sulfamethoxazole	34 ± 15	-	-	10.1	21 ± 7	1.9	140 ± 28
Sulfamonomethoxine	-	-	-	-	-	2.5	21 ± 6
Sulfapyridine	104 ± 47	-	-	0.4	630 ± 423	-	-
Tamsulosin	-	8.1	2 ± 2	-	-	20.4	1 ± 1
Temazepam	22 ± 5	8.2	25 ± 10	8.6	20 ± 11	6.4	112 ± 13
Terbutryn	14 ± 4	6.1	6 ± 1	10.4	7 ± 6	27.6	8 ± 4
Thiacloprid	-	-	-	18.8	17 ± 16	17.6	0.2 ± 0
Timolol	-	-	-	5.5	4 ± 6	21.5	1 ± 0.5
Tramadol	246 ± 45	-	-	5.2	13 ± 1	-	-
Trimethoprim	95 ± 20	-	-	3.9	122 ± 7	-	-
Valsartan	-	-	-	6.8	22 ± 29	2.2	22 ± 7
Venlafaxine	178 ± 33	-	-	2.8	117 ± 76	-	-
Verapamil	16 ± 2	3.7	11 ± 10	9	4 ± 2	22.5	3 ± 2
Warfarin	-	-	-	7.9	3 ± 1	4.5	4 ± 2

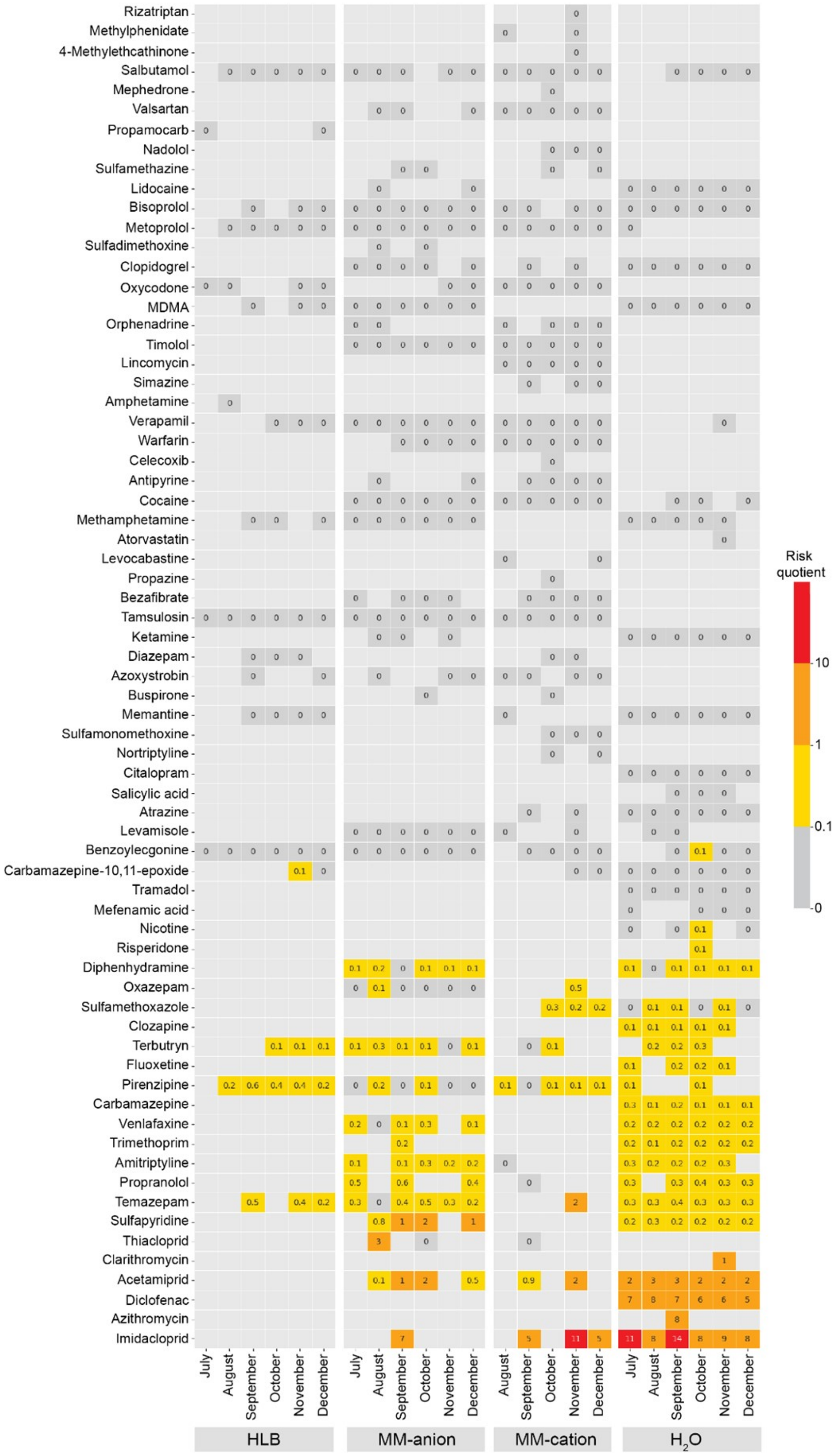

Figure S3. *Heatmap of the risk quotients (RQs) calculated for the HLB, MM-anion, and MM-cation passive sampler sorbents (for compounds with R_s values), and from the water CEC data for all compounds. Light grey tiles indicates compounds not quantified, compounds with a RQ of 0 are < 0.1.*

Table S7. Values used to calculate the toxic and effect units in *G. pulex* as per Eqn. 2 and 4. References for values are indicated.

Pesticide	Toxic units			Refs
	EC50 **(mg L^{-1})**	***BCF***	***$EC50_{int}$***	
Clothianidin	11.43	3	34,290	(Olker et al., 2022, EPA, 2015)
Imidacloprid	0.021	9	185	(Agatz et al., 2014, Miller et al., 2019)
Oxamyl	0.42	6	2,520	(Olker et al., 2022, Miller et al., 2019)
**PPCPs**	**Effect units**			
	$logD_{pH\ 7.4}$^{a}	***HtPC*** **(mg L^{-1})**	***PC_{crit}*** **(mg L^{-1})**	
Amlodipine	1.91	-	$7.0x10^{-3}$	(Fick et al., 2010)
Antipyrine	0.72	-	14	(Fick et al., 2010)
Benzatropine*	1.89	0.01	$3.2x10^{-3}$	(Schulz et al., 2012)
Carbamazepine	2.28	-	0.35	(Fick et al., 2010)
Citalopram	1.27	-	$1.4x10^{-4}$	(Fick et al., 2010)
Clarithromycin	2.37	-	$7.3x10^{-3}$	(Fick et al., 2010)
Clopidogrel*	4.21	0.001	$6.4x10^{-6}$	(Schulz et al., 2012)
Clozapine	2.71	-	0.32	(Fick et al., 2010)
Diclofenac	1.37	-	$4.6x10^{-3}$	(Fick et al., 2010)
Diphenhydramine	2.34	-	$2.0x10^{-3}$	(Fick et al., 2010)
Fluoxetine	1.75	-	$4.9x10^{-4}$	(Fick et al., 2010)
Ketoconazole	3.49	-	$4.3x10^{-3}$	(Fick et al., 2010)
Lidocaine	1.81	-	0.47	(Fick et al., 2010)
Lincomycin*	-0.44	1.5	24	(Nielsen and Gyrd-Hansen, 1998)
Nortriptyline	2.28	-	$5.6e^{-5}$	(Fick et al., 2010)
Propranolol	1.15	-	$2.0e^{-3}$	(Fick et al., 2010)
Risperidone	1.18	-	$1.3e^{-4}$	(Fick et al., 2010)
Sulfamethoxazole	-0.55	-	98	(Fick et al., 2010)
Sulfathiazole*	0.04	37.1	263	(Strum et al., 1978)
Trimethoprim	1.00	-	3.3	(Fick et al., 2010)
Venlafaxine	1.43	-	$6.1x10^{-3}$	(Fick et al., 2010(
Warfarin	0.31	-	0.19	(Fick et al., 2010)

[a] Predicted from SMILES using Percepta PhysChem Profiler; * Compounds for which the PC_{crit} was calculated from *logD* and H_TPC values.

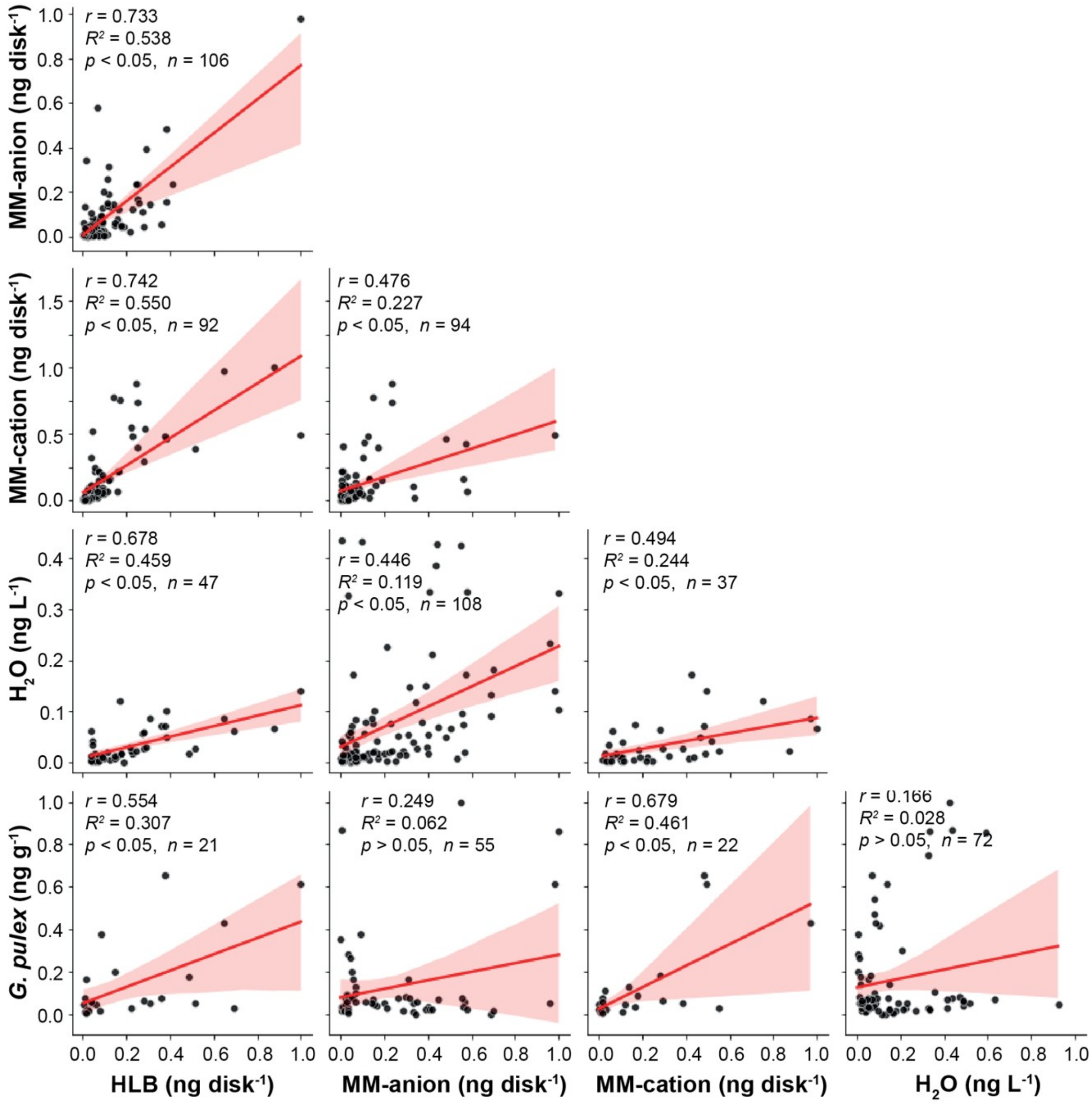

Figure S4. *Linear regressions of CEC concentrations between the different matrices with all data normalised between 1 and 0. The red trendline represents the line of best fit with a 95 % confidence interval (shaded).*

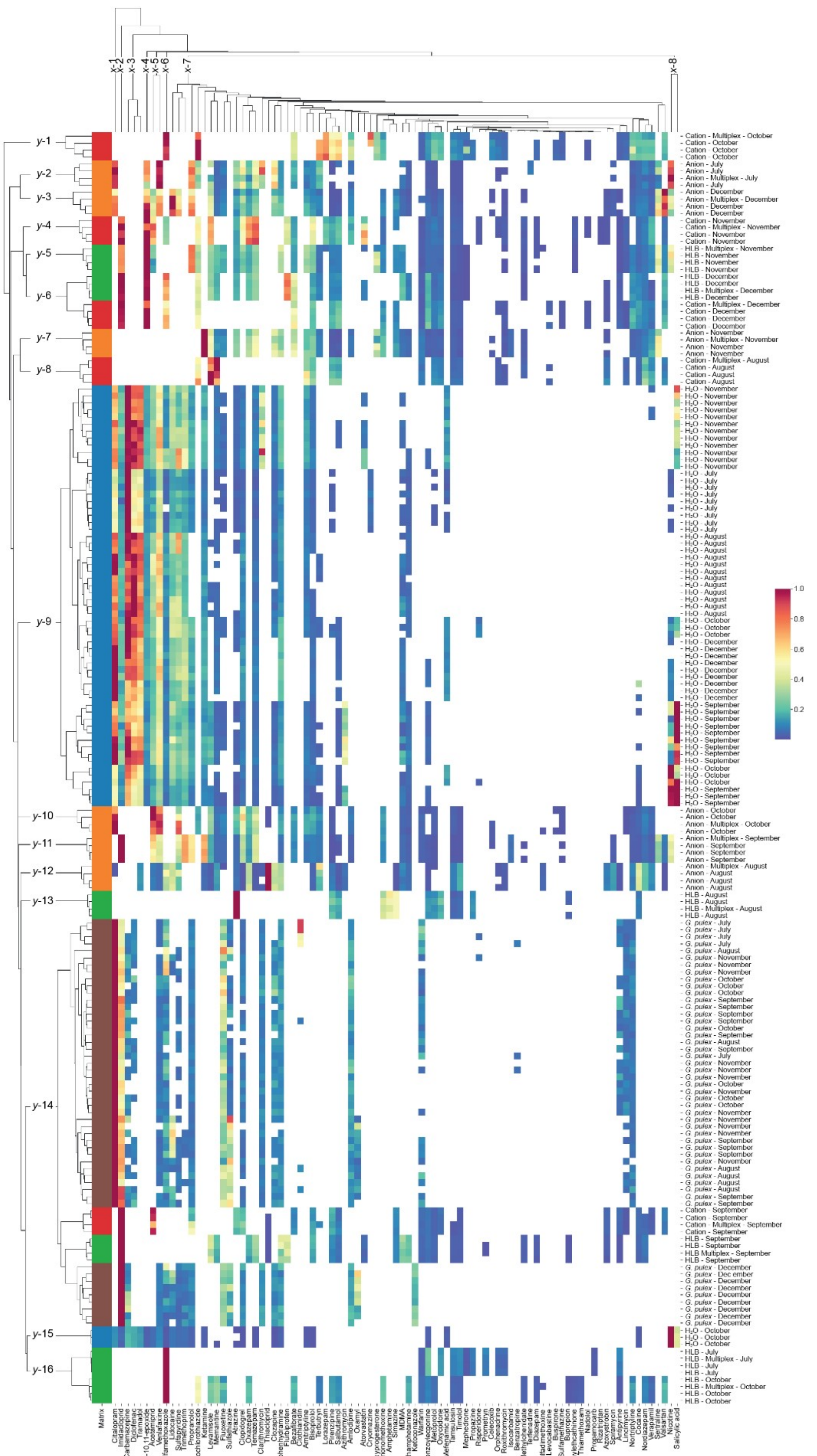

***Figure S5**. Hierarchical clustering analysis of all concentration data normalised, matrix indicated by left-hand side colour bar, red: MM-cation 3D-PSD, green: HLB 3D-PSD, orange: MM-anion 3D-PSD, blue: H2O, brown: G. pulex.*

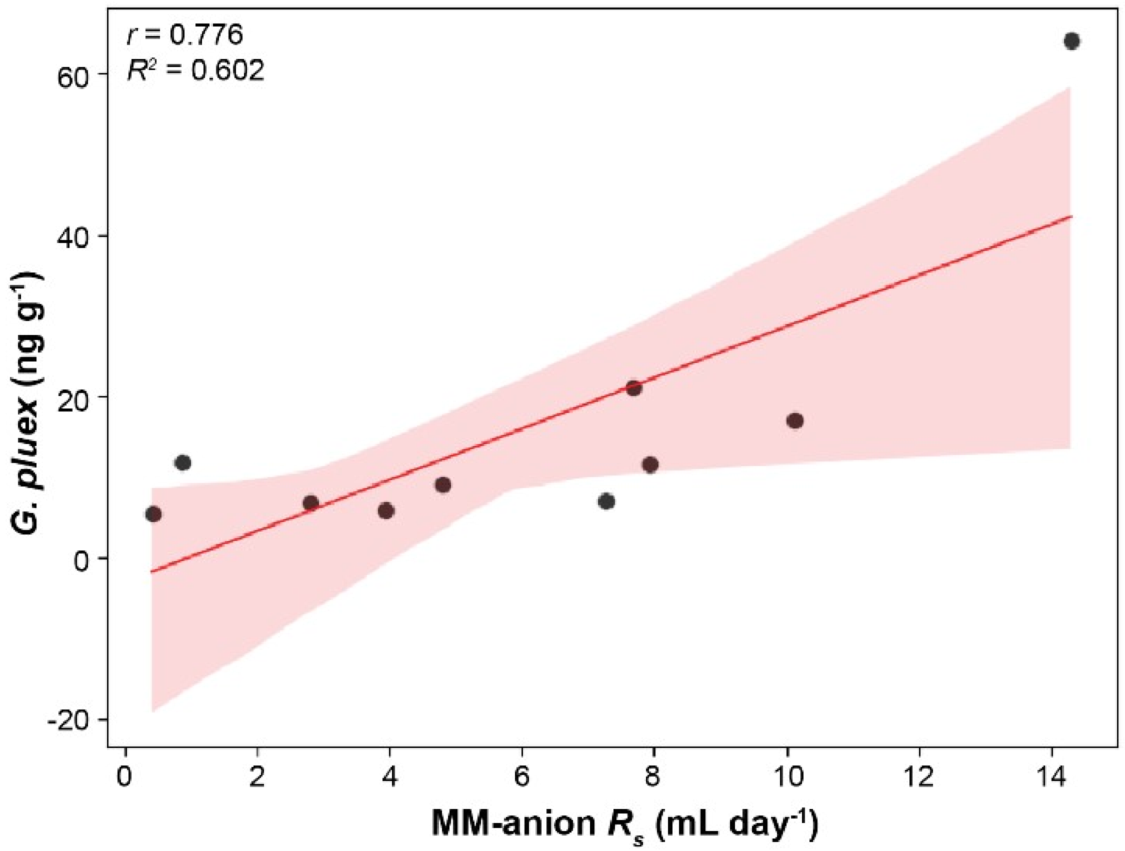

Figure S6. *Correlation between the MM-anion Rs values and the average contaminant concentration in the G. pulex across the whole study. The red trendline represents the line of best fit with a 95 % confidence interval (shaded).*

Table S8. Toxic units calculated from literature-derived data, values in bold exceed the -3 effect threshold.

Compounds	Calculated toxic units				
	Gammarid	**H_2O**	**HLB**	**MM-anion**	**MM-cation**
Azoxystrobin[1]	-4.2	-5.1	**-2.5**	**-2.5**	**-2.5**
Azoxystrobin[1]	-4.2	-4.7	**-2.1**	**-2.2**	**-2.2**
Azoxystrobin[1]	-3.9	-5.1	**-2.5**	**-2.5**	**-2.5**
Azoxystrobin[1]	-3.8	-4.4	**-1.9**	**-1.9**	**-1.9**
Imidacloprid[1]	**-2.2**	-3.4	-	**-0.4**	**-0.6**
Imidacloprid[1]	**-1.9**	-3.6	-	**-0.7**	**-0.8**
Imidacloprid[1]	**-1.7**	-3.7	-	**-0.8**	**-0.9**
Imidacloprid[2]	**-1.4**	-2.2	-	**0.7**	**0.6**
Propamocarb[3]	-6.6	-8.9	-7.4	-	-5.5
Propamocarb[3]	-6.5	-9.0	-7.5	-	-5.6
Thiacloprid[1]	-4.0	-6.8	-	-3.9	-4.0
Thiacloprid[1]	-3.9	-6.6	-	-3.8	-3.8

[1](Munz et al., 2018, 2017)
[2](Miller et al., 2019)
[3](Miller et al., 2021)

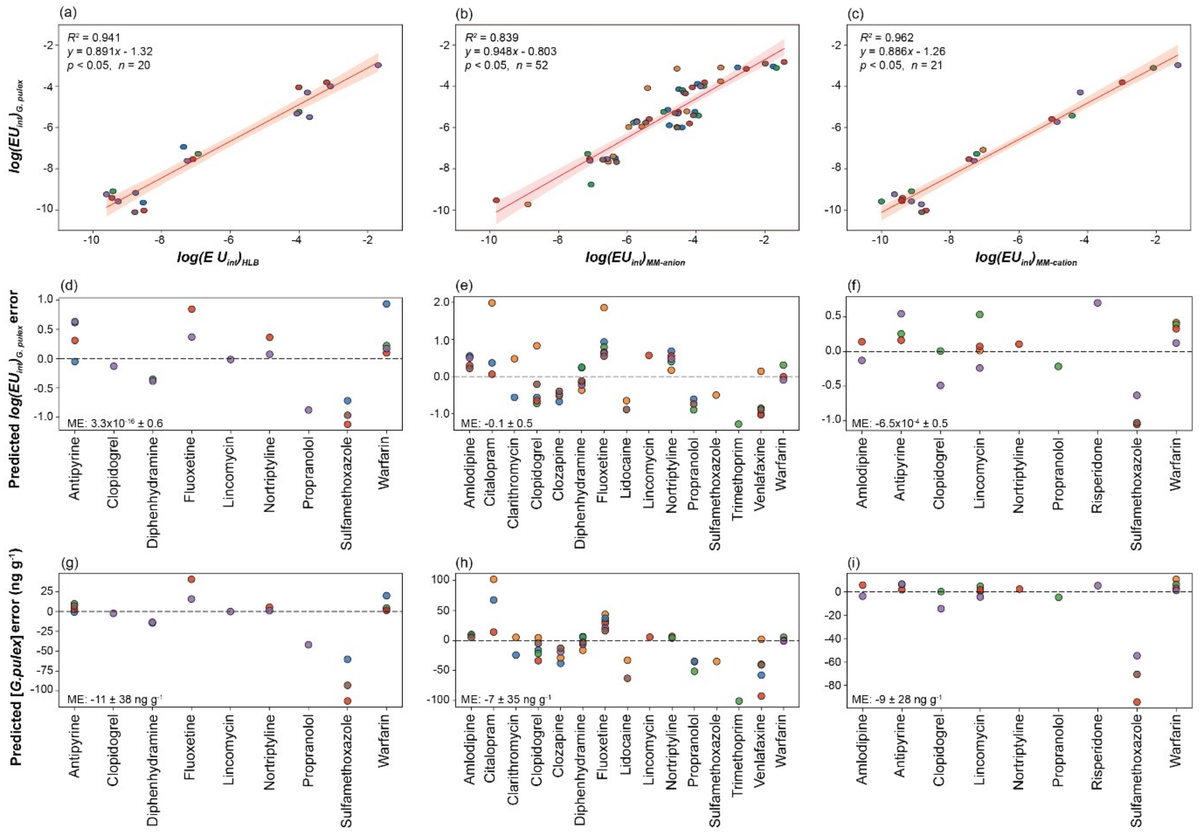

Figure S7. *(a) to (c), linear regression models for each passive sampler sorbent. (d) to (f), residual root of the predicted effect units in G. pulex using the corresponding model. (g) to (i), residual error of the predicted contaminant concentration using each of the models. Coloured dots indicate the month of sample collection, blue = July, orange = August, green = September, red = October, purple = November, and brown = December. The red trendline represents the line of best fit with a 95 % confidence interval (shaded).*

References

Agatz, A., Ashauer, R., Brown, C.D., 2014. Imidacloprid perturbs feeding of Gammarus pulex at environmentally relevant concentrations. Environ Toxicol Chem 33, 648–653. https://doi.org/10.1002/ETC.2480

Benson, D.A., Cavanaugh, M., Clark, K., Karsch-Mizrachi, I., Lipman, D.J., Ostell, J., Sayers, E.W., 2013. GenBank. Nucleic Acids Res 41. https://doi.org/10.1093/NAR/GKS1195

Castle, G.D., Mills, G.A., Bakir, A., Gravell, A., Schumacher, M., Townsend, I., Jones, L., Greenwood, R., Knott, S., Fones, G.R., 2018. Calibration and field evaluation of the Chemcatcher® passive sampler for monitoring metaldehyde in surface water. Talanta 179, 57–63. https://doi.org/10.1016/j.talanta.2017.10.053

Fick, J., Lindberg, R.H., Tysklind, M., Larsson, D.G.J., 2010. Predicted critical environmental concentrations for 500 pharmaceuticals. Regulatory Toxicology and Pharmacology 58, 516–523. https://doi.org/10.1016/J.YRTPH.2010.08.025

Miller, T.H., Bury, N.R., Owen, S.F., Barron, L.P., 2017. Uptake, biotransformation and elimination of selected pharmaceuticals in a freshwater invertebrate measured using liquid chromatography tandem mass spectrometry. Chemosphere 183, 389–400. https://doi.org/10.1016/j.chemosphere.2017.05.083

Miller, T.H., McEneff, G.L., Brown, R.J., Owen, S.F., Bury, N.R., Barron, L.P., 2015. Pharmaceuticals in the freshwater invertebrate, Gammarus pulex, determined using pulverised liquid extraction, solid phase extraction and liquid chromatography-tandem mass spectrometry. Science of the Total Environment 511, 153–160. https://doi.org/10.1016/j.scitotenv.2014.12.034

Miller, T.H., Ng, K.T., Bury, S.T., Bury, S.E., Bury, N.R., Barron, L.P., 2019. Biomonitoring of pesticides, pharmaceuticals and illicit drugs in a freshwater invertebrate to estimate toxic or effect pressure. Environ Int 129, 595–606. https://doi.org/10.1016/j.envint.2019.04.038

Miller, T.H., Ng, K.T., Lamphiere, A., Cameron, T.C., Bury, N.R., Barron, L.P., 2021. Multicompartment and cross-species monitoring of contaminants of emerging concern in an estuarine habitat. Environmental Pollution 270, 116300. https://doi.org/10.1016/j.envpol.2020.116300

Munz, N.A., Burdon, F.J., de Zwart, D., Junghans, M., Melo, L., Reyes, M., Schönenberger, U., Singer, H.P., Spycher, B., Hollender, J., Stamm, C., 2017. Pesticides drive risk of micropollutants in wastewater-impacted streams during low flow conditions. Water Res 110, 366–377. https://doi.org/10.1016/j.watres.2016.11.001

Munz, N.A., Fu, Q., Stamm, C., Hollender, J., 2018. Internal Concentrations in Gammarids Reveal Increased Risk of Organic Micropollutants in Wastewater-Impacted Streams. Environ Sci Technol 52, 10347–10358. https://doi.org/10.1021/ACS.EST.8B03632/ASSET/IMAGES/LARGE/ES-2018-03632E_0003.JPEG

Ng, K.T., Rapp-Wright, H., Egli, M., Hartmann, A., Steele, J.C., Sosa-Hernández, J.E., Melchor-Martínez, E.M., Jacobs, M., White, B., Regan, F., Parra-Saldivar, R., Couchman, L., Halden, R.U., Barron, L.P., 2020. High-throughput multi-residue quantification of contaminants of emerging concern in wastewaters enabled using direct injection liquid chromatography-tandem mass spectrometry. J Hazard Mater 398, 122933. https://doi.org/10.1016/j.jhazmat.2020.122933

Nielsen, P., Gyrd-Hansen, N., 1998. Bioavailability of spiramycin and lincomycin after oral administration to fed and fasted pigs. J Vet Pharmacol Ther 21, 251–256. https://doi.org/10.1046/J.1365-2885.1998.00131.X

OECD, 2019. Test No. 203: Fish, Acute Toxicity Test, OECD Guidelines for the Testing of Chemicals. Paris.

Olker, J.H., Elonen, C.M., Pilli, A., Anderson, A., Kinziger, B., Erickson, S., Skopinski, M., Pomplun, A., LaLone, C.A., Russom, C.L., Hoff, D., 2022. The ECOTOXicology Knowledgebase: A Curated Database of Ecologically Relevant Toxicity Tests to Support Environmental Research and Risk Assessment. Environ Toxicol Chem 41, 1520–1539. https://doi.org/10.1002/ETC.5324

Richardson, A.K., Irlam, R.C., Wright, H.R., Mills, G.A., Fones, G.R., Stürzenbaum, S.R., Cowan, D.A., Neep, D.J., Barron, L.P., 2022. A miniaturized passive sampling-based workflow for monitoring chemicals of emerging concern in water. Science of The Total Environment 839, 156260. https://doi.org/10.1016/J.SCITOTENV.2022.156260

Schulz, M., Iwersen-Bergmann, S., Andresen, H., Schmoldt, A., 2012. Therapeutic and toxic blood concentrations of nearly 1,000 drugs and other xenobiotics. Crit Care 16, R136. https://doi.org/10.1186/CC11441

Storey, A.W., Edward, D.H.D., Gazey, P., 1991. Surber and kick sampling: a comparison for the assessment of macroinvertebrate community structure in streams of south-western Australia. Hydrobiologia 211, 111–121. https://doi.org/10.1007/BF00037367/METRICS

Strum, J.D., Colaizzi, J.L., Goehl, T.J., Jaffe, J.M., Pitlick, W.H., Shah, V.P., Poust, R.I., 1978. Bioavailability of Sulfonamide Suspensions I: Dissolution Profiles of Sulfamethizole Using Paddle Method. J Pharm Sci 67, 1399–1402. https://doi.org/10.1002/JPS.2600671018

Taylor, A.C., Mills, G.A., Gravell, A., Kerwick, M., Fones, G.R., 2021. Passive sampling with suspect screening of polar pesticides and multivariate analysis in river catchments: Informing environmental risk assessments and designing future monitoring programmes. Science of The Total Environment 787, 147519. https://doi.org/10.1016/J.SCITOTENV.2021.147519

Untergasser, A., Cutcutache, I., Koressaar, T., Ye, J., Faircloth, B.C., Remm, M., Rozen, S.G., 2012. Primer3—new capabilities and interfaces. Nucleic Acids Res 40, e115. https://doi.org/10.1093/NAR/GKS596

Vrana, B., Allan, I.J., Greenwood, R., Mills, G.A., Dominiak, E., Svensson, K., Knutsson, J., Morrison, G., 2005. Passive sampling techniques for monitoring pollutants in water. TrAC - Trends in Analytical Chemistry 24, 845–868. https://doi.org/10.1016/j.trac.2005.06.006
